# Inverse optimal control with time-varying objectives: application to human jumping movement analysis

**DOI:** 10.1038/s41598-020-67901-x

**Published:** 2020-07-07

**Authors:** Kevin Westermann, Jonathan Feng-Shun Lin, Dana Kulić

**Affiliations:** 1 University of Waterloo, Waterloo, Canada; 2 Monash University, Melbourne, Australia

**Keywords:** Biomedical engineering, Motor control

## Abstract

Analysis of complex human movements can provide valuable insights for movement rehabilitation, sports training, humanoid robot design and control, and human–robot interaction. To accomplish complex movement, the central nervous system must coordinate the musculo-skeletal system to achieve task and internal (e.g., effort minimisation) objectives. This paper proposes an inverse optimal control approach for analysing complex human movement that does not assume that the control objective(s) remains constant throughout the movement. The movement trajectory is assumed to be optimal with respect to a cost function composed of the sum of weighted basis cost functions, which may be time varying. The weights of the cost function are recovered using a sliding window. To illustrate the proposed approach, a dataset consisting of standing broad jump to targets at three different distances is collected. The method can be used to extract control objectives that influence task success, identify different motion strategies/styles, as well as to observe how control strategy changes during the motor learning process. Kinematic analysis confirms that the identified control objectives, including centre-of-mass takeoff vector and foot placement upon landing are important to ensure that a given participant lands on the target. The dataset, including nearly 800 jump trajectories from 22 participants is also provided.

## Introduction

Investigating how humans perform dynamic movements is important for many applications. Analysis of motor control behaviour can be useful for movement rehabilitation and sports training, in order to help those who are injured or otherwise limited in their mobility to attain or reattain efficient and effective movement^1–3^. Human motor control strategies can also be used to replicate human-like movement on humanoid and other robot platforms^4,5^, and machines that interact with humans in social environments benefit from a model of human behaviour to predict their future actions^6–8^.

The theory of task optimisation hypothesises that humans typically optimise a set of criteria when moving, and the central nervous system translates these high-level goals into low-level motor control behaviour^9,10^. The focus of this paper is to develop a methodology for analysing dynamic human movement by identifying the features or characteristics which are crucial to task success, and understanding optimal motor control behaviour required to complete the movement. One important characteristic of complex movements is the possibility that the control objectives may not be constant throughout the movement. For example, when trying to jump as far as possible, the jumper might first try to optimise the centre of mass (CoM) trajectory at takeoff^11–13^, then achieve a preferred posture during the flight phase, and finally minimise impact on landing.

This paper investigates an approach for identifying the control objectives from human movement data during complex movements. The proposed approach is based on a windowed inverse optimal control (IOC) approach^14^. Many prior works in IOC^4,15^ assume that the cost function optimised within each pre-defined segment is constant, while the windowed approach allows this assumption to be relaxed, thus eliminating the need to pre-segment the motion trajectory or assume that the underlying cost function is constant throughout the motion. In this paper, we extend the prior work by applying the IOC analysis to a full body model, and a large data set of nearly 800 movement trajectories from 22 participants. The full dataset is also provided as part of this paper.

**Fig. 1:**
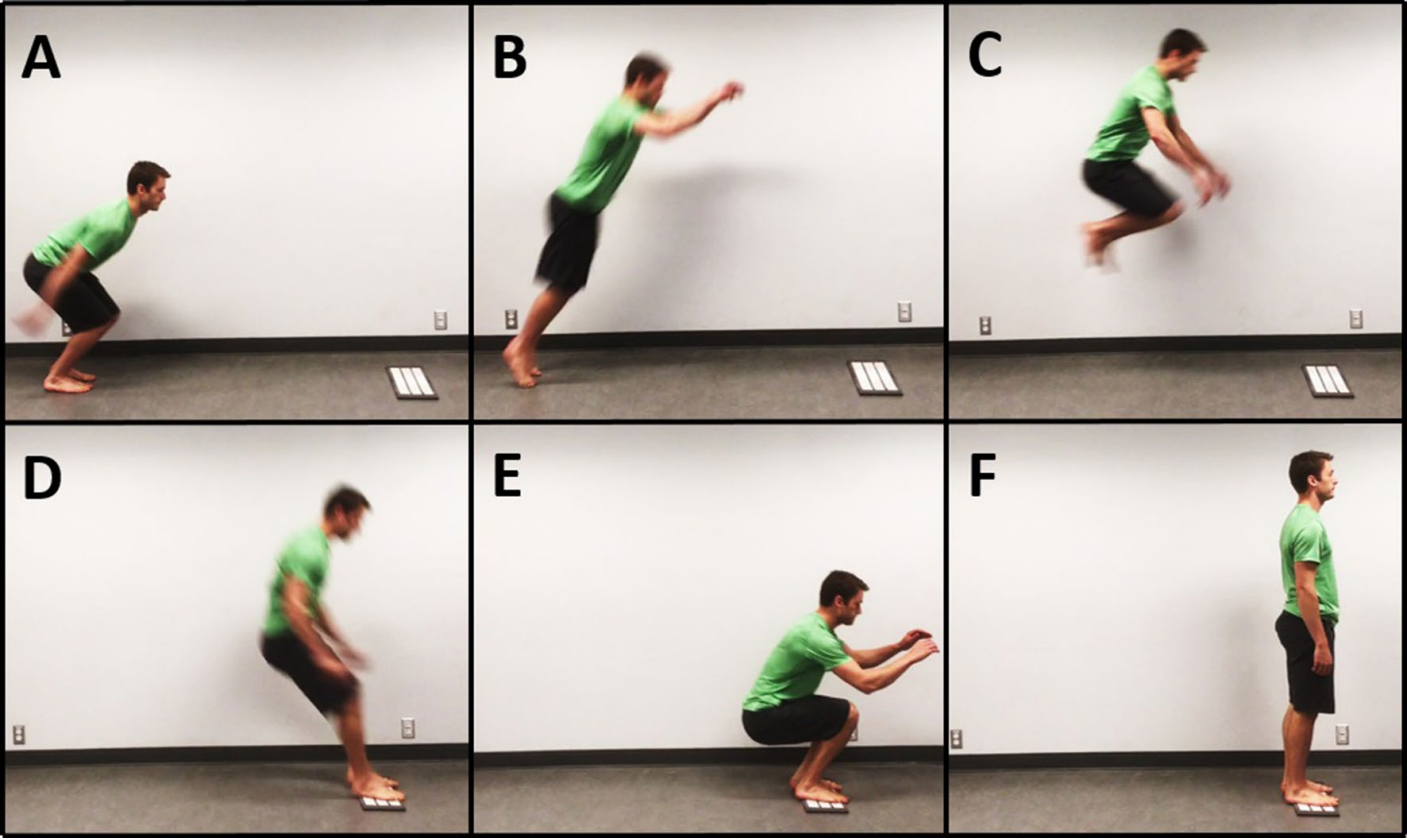
Phases of the standing broad jump: (**A**) start of takeoff phase, (**B**) takeoff (**C**) flight, (**D**) ground contact at landing, (**E**) landing, and (**F**) finish. The white platform at the landing location is used to indicate the jump target.

Jumping to a target (i.e. aiming to land in a pre-determined location) was selected as an example dynamic movement (Fig. 1). Jumping motions are highly dynamic and require full-body coordination to complete successfully. The task also has a clear task objective and metric of success, which facilitates evaluating task performance.

The rest of this paper is organised as follows: in Sect. 2, prior work in jumping biomechanics and control is reviewed. The data collection protocol and IOC analysis procedure are described in Sect. 3 while additional details about the collected dataset and experimental results can be found in the Supplementary Materials S1. Next, Sect. 4 summarises the kinematic analysis identifying the key task objectives, namely the takeoff CoM velocity vector and the foot placement, and how they impact landing success. Section 5 describes the results of the IOC analysis and uses the results to identify similarities and differences of control priorities during the jump trajectory, and different jumping styles. Section 6 summarises the findings. The Supplementary Material S1 contains additional detail on the data collection methodology, and instructions for accessing the jumping dataset files.

## Related work

Early human jumping research typically aimed to determine the kinematic and biomechanical patterns in the leg required for jumping, usually by analysing image and force plate data from human motion data collection. Robertson and Flemming^16^ determined that hip, knee and ankle extension during the takeoff phase of jumping contribute different percentages of propulsion for vertical and standing broad jumping, and that extensor muscle groups at these joints contract simultaneously to produce the leg extension. Bosco et al.^17^ observed greater mechanical efficiency of vertical jumping when incorporating pre-stretching of the extensor muscles at the knee during the takeoff phase, as well as increased efficiency when the knee started from a more extended position. Wakai and Linthorne^18^ analysed the standing broad jump performed at various takeoff angles and determined that an angle between $19^{\circ }$ and $27^{\circ }$ to the horizontal was the optimum takeoff angle to achieve maximum distance. Ashby^19^ compared motion trajectories of jumpers using their arms normally and constraining their arms to the torso. Jumpers travelled 21% farther when able to use their arms, and were observed to swing their arms to counter the forward angular momentum produced by the legs and torso, enabling greater overall linear momentum to be generated at takeoff. Without the influence of the arms, a jumper must eliminate excessive forward body rotation before entering the flight phase.

Research has also investigated the factors that influence landing stability. Seegmiller and McCaw^20^, and Wikstrom et al.^21^ found that the magnitude and direction of the ground reaction force upon landing was a major factor. Larger impacts typically lead to poor stability and higher injury rate. Jumps in the forward direction tend to be easier to land without losing balance than diagonal or lateral jumps, but also led to higher vertical impact force relative to the participant’s body mass. Joint range of motion and torque also influence landing stability. McKinley and Pedotti^22^ found that expert jumpers land with greater variance in ankle range of motion and joint torque when landing on different surface rigidity, giving them a greater ability to adjust to different landing conditions than novices. In addition to ankle kinematics, knee motion also plays a factor in landing stability. Ford et al.^23^ examined knee valgus motion during drop jumps and found that females exhibit significantly larger knee valgus motion on impact than males due to having less musculature to control the joint torques in the lower body during an athletic manoeuvre, leading to less joint control on landing and higher rate of knee ligament injuries in females.

Dynamic model approaches have been proposed to analyse the jump motion. Özgüven and Berme^24^ used force plates to measure impact forces during vertical and depth jump landings, and predicted the spring, damper and inertial parameters of a 2 degree of freedom (DoF) model based on experimental and anthropological data. Pandy et al.^11^ developed a simple four link planar model and used an optimisation algorithm to control the model to perform a maximum height jump. Guihard and Gorce^25^ expanded on the work of Pandy by developing a controller for vertical jumping for use with biped control of planar, three link rigid body legs. Cheng et al.^26^ investigated the role of arm motion in vertical jumping with a dynamic five link model, finding the optimal joint activation timing to result in the greatest jump height. The results were used to explain the validity of multiple human movement energy generation theories. Meghdari and Aryanpour^12^ developed a full body, sagittal plane dynamic jumping model framework that could calculate and reproduce the kinematics and dynamics of real jump trajectories recorded through image data. Farahani et al.^13^ also proposed a full body, sagittal dynamic jumping model that computes the dynamics of vertical jumps from motion capture data.

The results of these simulation studies indicate that optimised trajectories based on simplified kinematic and dynamic models show good correspondence with observed human trajectories, providing support for the hypothesis that the central nervous system generates movement by optimising some criteria^10^. This hypothesis gave rise to IOC methods, where a human trajectory is analysed to estimate the underlying cost function optimised to generate the trajectory. While few IOC works have examined jumping tasks, numerous IOC efforts have been applied to other task-space based motions. In this type of approach, the cost function is hypothesised to be formed as a weighted sum of basis cost terms, and the objective is to estimate the set of weights, describing the relative influence of each of the basis cost terms. Berret et al.^3^, Albrecht et al.^27^, and Sylla et al.^28^ examined hand reaching, contact, and interaction tasks using the bi-level approach to determine the relevant cost functions, where an optimisation loop determines the optimal trajectory given a set of weights, while a second loop calculates the weights that minimise the error to the observed trajectory. Lin et al.^14^ and Panchea et al.^29^ analysed the squat exercises and overhead arm tasks by applying the inverse Karush–Kuhn–Tucker (KKT) method, where the KKT optimality condition was applied to calculate the cost function weights that minimise the optimality condition violation residuals. In robotics applications, Finn et al.^30^ and Englert and Toussaint^31^ utilise IOC to extract the cost function for common task-driven human activities such as placing dishes into dish rack and opening doors, for the purpose of reproducing the task by a robot, while Mainprice et al.^32^ use IOC to learn human movement during pick and place tasks to facilitate human-robot collaborative tasks.

For jumping tasks, Maldonado et al.^33^ analysed parkour jumping to a target task by estimating the relative importance of cost functions corresponding to take-off force, landing posture, impact loading rate, and fall avoidance. However, their work examined only seven expert jumpers over a small set of cost functions during the take-off and landing phase. In this work, we analyse the data from 22 participants with varying jumping expertise, and perform a comprehensive kinematic and control-based analysis over the full jumping trajectory.

## Methods

### IOC Protocol

The inverse KKT IOC approach^14^ was used to determine the motor control tasks that jumpers prioritised throughout the jumping motion, based on the joint trajectories calculated from the pose estimation data processing. This analysis was performed by assuming that the observed human motion is generated by an optimal controller, which optimises a cost function consisting of a sum of weighted basis functions $J_i$ (Eq. 1). The basis functions represent different motor control objectives that the central nervous system may be optimising to perform a given human motion^3,10^:

1 $$\begin{aligned} J(\mathbf {x}) = \sum _{i=0}^{n_{ct}} w_i J_{ct,i}(\mathbf {x}) \end{aligned}$$

where $w_i$ are the weights representing the relative importance of each basis function $J_i$.

In the IOC setting, we have observed a trajectory described by the joint positions, velocities, and accelerations $\mathbf {x_{opt}} = [\mathbf {q_{opt}},  {\dot{\mathbf{q}}_{opt}},  {\ddot{\mathbf{q}}_{opt}}]$ that were optimised according to Eq. 1. Our objective is to recover an estimated weight vector $\hat{w}_{i}$ from the observed trajectory. We solve this problem by considering the KKT conditions, which are a set of necessary conditions for a solution to a non-linear optimisation problem to be considered optimal. To recover the weights, the KKT Lagrangian $L(\mathbf {x})$ and its gradient $\mathbf {\nabla _x} L(\mathbf {x})$ are defined as:

2 $$\begin{aligned} L(\mathbf {x})&= \sum _{i=0}^{n_{ct}} \hat{w}_{i} J_{ct, i}(\mathbf {x_{obs}}) + \sum _{j=0}^{n_{h}} \lambda _j h_j(\mathbf {x_{obs}}) \nonumber \\ \mathbf {\nabla _x} L(\mathbf {x})&= \sum _{i=0}^{n_{ct}} \hat{c}_{i} \mathbf {\nabla _x} J_{ct, i}(\mathbf {x_{obs}}) + \sum _{j=0}^{n_{h}} \lambda _j \mathbf {\nabla _x} h_j(\mathbf {x_{obs}}) \end{aligned}$$

where the partial differential of the gradient $\mathbf {\nabla _x}$ is calculated with respect to the state variables $\mathbf {x}$, $\mathbf {\lambda }$ are the Lagrangian multipliers on constraints $h(\mathbf {x})$, and $\mathbf {Q_{obs}}$ is constructed from the spline representation of the trajectory to reduce computation time. The condition that must be met to ensure optimality is:

3 $$\begin{aligned} \mathbf {\nabla _x} L(\mathbf {Q_{obs}})&= 0 \end{aligned}$$

If it is assumed that the system is not strictly optimal, but rather only approximately optimal, then Eq. 3 is minimised but is not strictly zero:

4 $$\begin{aligned} \min _{ {\hat{\mathbf{c}}}, \mathbf {\lambda }} \mathbf {\nabla _x} L(\mathbf {Q_{obs}}) \in  {\hat{\mathbf{c}}} \ge 0 \end{aligned}$$

Since the KKT equations are linear with respect to the unknown variables $\mathbf {z} = [{\hat{\mathbf {c}}}, \mathbf {\lambda }]$, Eq. 4 can be written as a least square problem in the form of $\mathbf {A} \mathbf {z}$ = 0, where $\mathbf {A}$ is the gradient. $\mathbf {z}$ can then be solved as a least-squares problem.

The inverse KKT is applied to a sliding window over the full trajectory. For all timesteps with an overlapping window, the weights generated at that timestep are averaged together to estimate the weight for that timestep (Fig. 4). Those terms with the highest weights in each input trajectory window are assumed to correspond with the motor control tasks being optimised during that window. These weight trajectories were then analysed by manually separating the jumping data into specific groups designed to identify motor control differences related to specific jump features.

A major limitation of this method is that a global minimum cannot be guaranteed due to factors including the non-linearity of the underlying cost functions, loss of rank of the gradient matrix, or non-observability of certain states and cost functions over parts of the trajectory. This is a weakness that the inverse KKT method shares with other common IOC methods, such as the bi-level approach^4^.

### IOC cost function design

Human motor control tasks were hypothesised to form the cost terms in the IOC cost function. First, control tasks specific to the jumping motion were hypothesised, including task space position, velocity and acceleration of the CoM and toe positions of the body, relative to the global frame (with the origin located at the target position) and each other (i.e. velocity of CoM relative to velocity of the toes). The CoM and toe trajectories, and their relative positions to the target, were selected as the jumping-specific cost terms based on the findings of the kinematic trajectory analysis detailed in Sect. 4.

Second, control tasks that have been previously identified as relevant for human motion^3^ were hypothesised, including the acceleration and jerk of the joints, joint torques and their derivatives, kinetic energy, and power of the system.

Finally, the functions for joint acceleration and jerk, joint torques and their derivatives, and joint power were split into separate cost terms for the arms (shoulders and elbows), legs (hips, knees and ankles), and torso (pelvis and lower back rotation). These sections of the body move with different velocities and have different roles in the jumping motion: the arms move fast, contribute moderate momentum generation and help landing stability; the legs move with moderate speed and are the primary contributor to momentum generation and landing impact absorption; and the torso moves slowly and is the anchor for the arms and legs^12^. The differing velocities and contributions of body limbs throughout the jump phases warrant separate cost functions^34^. Kinetic energy was not separated since this value includes the energy the entire body has when travelling through the air during the flight phase.

The cost terms in the IOC cost function must be independent to prevent singularities during the inverse KKT solver matrix calculations^14^. All jumping-specific cost terms in the task space were divided into forward horizontal (global X axis) and vertical (global Z axis) directions to avoid dependency between different cost terms.

In total, 40 different cost terms were hypothesised. Initial testing revealed that several of the cost functions were recovered with zero weights and did not contribute to the minimisation of the KKT residuals, leaving a final list of 17 cost terms (Table 1).

**Table 1:** The final set of IOC cost terms, summed over $n_a$ DoFs or $n_b$ bodies, and *T* time (where *T* is the length of each window for which IOC is performed).

Cost weight	Cost term equation	Description
$w_{KE}$	$$J_{KE} = \begin{aligned} \sum _{a}^{n_a} \sum _{t}^{T} {\dot{q}_{a, t}}^T M(q) \dot{q}_{a, t} \end{aligned}$$	Kinetic energy of model
$w_{CoM-Z}$	$$J_{CoM-Z} = \begin{aligned} \sum _{b}^{n_b} \sum _{t}^{T} m_b c_{Z,b, t} \end{aligned}$$	CoM height
$w_{CoM-dZ}$	$$J_{CoM-dZ} = \begin{aligned} \sum _{b}^{n_b} \sum _{t}^{T} (m_b \dot{c}_{Z,b, t})^2 \end{aligned}$$	CoM vertical velocity
$w_{Toe-dZ}$	$$J_{Toe-dZ} = \begin{aligned} \sum _{t}^{T} \dot{x}_{Toes-Z, t}^2 \end{aligned}$$	Toe vertical velocity
$w_{Toe-dX}$	$$J_{Toe-dX} = \begin{aligned} \sum _{t}^{T} \dot{x}_{Toes-X, t}^2 \end{aligned}$$	Toe forward velocity
$w_{CoMToe-dX}$	$$J_{CoMToe-dX} = \begin{aligned} \sum _{b}^{n_b} \sum _{t}^{T} (m_b \dot{c}_{X,b, t})^2 - J_{Toe-dX} \end{aligned}$$	CoM forward velocity relative to toe
$w_{ddq-tor}$	$$J_{ddq-tor} = \begin{aligned} \sum _{a}^{n_{a,tor}} \sum _{t}^{T} \ddot{q}_{a, t}^2 \end{aligned}$$	Torso: joint acceleration
$w_{dddq-tor}$	$$J_{dddq-tor} = \begin{aligned} \sum _{a}^{n_{a,tor}} \sum _{t}^{T} \dddot{q}_{a, t}^2 \end{aligned}$$	Torso: joint jerk
$w_{\tau -tor}$	$$J_{\tau -tor} = \begin{aligned} \sum _{a}^{n_{a,tor}} \sum _{t}^{T} \tau _{a, t}^2 \end{aligned}$$	Torso: joint torque
$w_{dq\tau -tor}$	$$J_{dq\tau -tor} = \begin{aligned} \sum _{a}^{n_{a,tor}} \sum _{t}^{T} (\dot{q}_{a, t} \tau _{a, t})^2 \end{aligned}$$	Torso: joint angular power
$w_{ddq-arm}$	$$J_{ddq-arm} = \begin{aligned} \sum _{a}^{n_{a,arm}} \sum _{t}^{T} \ddot{q}_{a, t}^2 \end{aligned}$$	Arms: joint acceleration
$w_{dddq-arm}$	$$J_{ddqq-arm} = \begin{aligned} \sum _{a}^{n_{a,arm}} \sum _{t}^{T} \dddot{q}_{a, t}^2 \end{aligned}$$	Arms: joint jerk
$w_{\tau -arm}$	$$J_{\tau -arm} = \begin{aligned} \sum _{a}^{n_{a,arm}} \sum _{t}^{T} \tau _{a, t}^2 \end{aligned}$$	Arms: joint torque
$w_{dq\tau -arm}$	$$J_{dq\tau -arm} = \begin{aligned} \sum _{a}^{n_{a,arm}} \sum _{t}^{T} (\dot{q}_{a, t} \tau _{a, t})^2 \end{aligned}$$	Arms: joint angular power
$w_{ddq-leg}$	$$J_{ddq-leg} = \begin{aligned} \sum _{a}^{n_{a,leg}} \sum _{t}^{T} \ddot{q}_{a, t}^2 \end{aligned}$$	Legs: joint acceleration
$w_{dddq-leg}$	$$J_{dddq-leg} = \begin{aligned} \sum _{a}^{n_{a,leg}} \sum _{t}^{T} \dddot{q}_{a, t}^2 \end{aligned}$$	Legs: joint jerk
$w_{\tau -leg}$	$$J_{\tau -leg} = \begin{aligned} \sum _{a}^{n_{a,leg}} \sum _{t}^{T} \tau _{a, t}^2 \end{aligned}$$	Legs: joint torque

*M* denotes the inertia matrix, *m* denotes the mass of a single link in the kinematic model, *c* denotes the Cartesian position of the CoM. Note that cost term 6 is the difference in forward velocity between the CoM and the toe. This term provided a lower KKT residual error than CoM forward velocity alone (relative to the global frame).

### IOC weights clustering analysis

In addition to the manual analysis, we were interested in seeing if the recovered weights can be used to automatically group together similar motor control patterns. Unsupervised clustering was performed on the IOC weight trajectory data for each jump using the kmeans++ algorithm^35^. Kmeans++ was used to cluster the recovered cost term weight trajectories, where weight trajectories from one jump formed one observation.

### Data collection

Twenty two participants (13 M, 9 F) with $\mu _{age} = 26$ without any serious injuries in the previous 6 months were recruited. Each participant completed multiple jumps to a pre-defined target location indicated by a foam mat anchored to the ground. All jumping motions in the study were standing broad jumps to a target, starting with both feet on the ground, jumping with both feet leaving the ground at approximately the same time, and then landing with both feet contacting the ground at approximately the same time (Fig. 1). Shoes were worn for all jumps in the experiment. A participant’s maximum standing broad jump distance was used to scale the landing target distances to the physical abilities of the jumper. Participants then completed 12 jumps in two groups of six to each to three different target distances, set at 55%, 70%, and 85% of the participant’s maximum jump distance, for a total of 36 jumps per participant.

The data was collected with eight Motion Analysis motion capture cameras using 30 markers at 200 Hz. All collected data were included in the analysis with the exception of Participant 1, due to the large number of marker occlusions and jumps beyond the camera range. The camera field of view was adjusted after Participant 1. For additional details on the data collection protocol and motion capture set up, please see the Data Collection section in the Supplementary Materials S1.

This experiment was approved by the University of Waterloo Research Ethics Board, in accordance with the Declaration of Helsinki, and written informed consent was obtained from all participants, including the participant shown in Fig. 1. Furthermore, the participant in Fig. 1 has provided explicitly permission to include this figure in this manuscript as a possible identifying image in an online open-access publication.

An extended Kalman filter approach^36^ was used to transform the motion capture marker position data into joint trajectories using a standard 3D 35 DoF kinematic model^37^ with anthropometric hip placements^38^ and additional prismatic joints at the shoulder to improve scapula modelling (Fig. S4 in the Supplementary Materials S1). A simplified 2D 14 DoF model, consisting of only the sagittal revolute joints (Fig. S4, ten red joints denoted by cylinders) and the floating base (three prismatic joints, one revolute joint), was used for IOC analysis. Each of the 12 jumps per distance per participant were temporally aligned by shifting all trajectories such that foot contact post-jump occurs at the same timestep. The time points of takeoff and landing were determined by manually inspecting each trajectory to determine when the motion capture markers on the foot left or contacted the ground, respectively. Note that this manual segmentation of the trajectory is not used for generating the IOC results, it is only used for the kinematic analysis in Sect. 4, and for comparing the IOC results to the kinematic analysis in Sect. 5.

### Jump evaluation

Each jump was assigned an evaluation grade by the experimenter during the data collection based on landing location relative to goal or if corrective motion was needed to prevent falling after landing, serving as a metric to evaluate each jump’s success. The jumps were classified into one of the following categories.

**B**: landed to the back of the target with some part of the foot touching the floor; unsuccessful jump
**SB**: slightly back of the middle of the foam mat target, may have needed balance corrections to stabilise; successful jump
**P**: landed in the middle of the foam mat target with control; perfect jump
P*: landed in the middle of the foam mat target, needed balance corrections to stabilise; perfect landing but lacking control
**SF**: landed slightly forward of the middle of the foam mat target, may have needed balance corrections to stabilise; successful jump
**F**: landed forward of the target with some part of the foot touching the floor; unsuccessful jump

The jumpers with the highest jump success rates during the experiment were considered experts, denoted by achieving perfect jumps on over 80% of the attempts (8 of 21 participants). All jumpers with prior experience in jumping to a target also exceeded the 80% threshold (three of eight expert participants). The mean number of successful jumps from all expert participants was 27.4/36 (76%), while the novice participants had a mean of 17.8/36 (49%) successful jumps. The dataset is summarised in Table 2, while a more detailed breakdown can be found in Table S8 in the Supplementary Materials S1.

**Table 2:** Number of jumps in dataset in each jump grade category, for all participants.

Jump grade	Abbreviation		Target 1		Target 2		Target 3	
			Set 1	Set 2	Set 1	Set 2	Set 1	Set 2
Back	B	Blue	1	1	1	3	6	4
Slightly back	SB	Purple	8	6	10	9	16	15
Perfect	P	Green	71	92	75	87	64	72
Perfect, balance correction	P*	Green, dashed	33	18	25	17	22	20
Slightly Forward	SF	Yellow	9	6	10	4	11	8
Forward	F	Red	4	3	5	6	7	7

The jump targets are noted as 55%, 70%, and 85% of the participant’s maximum jump. The colours denote the coding legend used thoughout this paper.

### IOC algorithm parameters

The sliding window IOC algorithm utilised a spline reconstruction knot concentration of 40 knots per second, with a sliding window length of 0.2 s. This corresponds to one knot every five timesteps at 200 Hz, which is just above the minimum four timesteps required to calculate jerk numerically. This minimum knot concentration was selected so that the cost function value corresponding to each knot will not overlap with another knot. The window width was selected in order to capture the flight phase of the shortest jumps, and served as a compromise between a tighter window that would have fewer knots to construct the spline, and a larger window or knot spacing which would decrease the resolutions of the recovered weights due to considering too much of the fast moving jumping trajectory in a single window. A window shift of ten frames was used to balance between computation time and the need to keep the start and the end of the window on a knot point.

Cost terms were normalised to reduce the impact of varying unit magnitudes by calculating the range of the cost function over the flight phase of a trajectory, and using that range as the fixed normalisation coefficient for each sliding window. Normalising over the entire trajectory, the takeoff phase only, or the landing phase only was also investigated. In these cases the recovered IOC weight trajectories were typically dominated by a single cost term, with most or all other terms providing a negligible contribution. This result suggests that normalisation over the initial calibration motions and/or ground contact phases do not provide appropriate trajectory data to recover the weights during the flight phase, the most dynamic portion of the jump.

For the kmeans++ algorithm, cluster indices were compiled using between 2 and 20 clustering groups, and then the cluster results were compared. Clustering was performed multiple times for each number of groups to see how repeatable the classification of the observations was, since the kmeans++ algorithm is based on randomly initialised centroids^35^.

After isolating interesting variables using visual analysis or clustering, statistical analysis was then performed to determine if any of the identified differences were statistically significant. Results were considered statistically significant at $p <0.05$. All statistical analysis was done using MATLAB 9.6. All statistical input and output parameters is included in the Supplementary Statistical Analysis section in the Supplementary Materials S1.

## Kinematic analysis

A kinematic analysis was first performed to identify features relevant to jump success. During the jumping task, the body can be thought of as a ballistic projectile, therefore two key task objectives are to control the CoM velocity and direction at takeoff and foot placement prior to and at landing. A successful jump requires the CoM to be projected into an acceptable ballistic trajectory range during the jumping motion, which is achieved by adjusting the CoM takeoff velocity. At landing, the feet must be placed on the target and the legs controlled to slow down the body’s momentum and stabilise the CoM over the feet. It was observed from the collected dataset that some trajectories do not start with an appropriate CoM velocity to land on the target by simple ballistic kinematics, but the feet placement timing can be adjusted to still land on the target.

Variations in technique were observed between participants, such as leg joint angle trajectory during takeoff and landing, various methods of using the arms to generate momentum and regain balance through the jump, and the height of the torso and feet during the flight phase. Expert jumpers were observed to have more consistent CoM takeoff velocity and better foot placement control. Novice jumpers were observed to improve their takeoff velocity consistency after only a few jumps, however, accurate and adaptable foot placement control appears to take more time and experience to develop.

### Center of mass trajectories on takeoff

For a jump to a desired target to be successful, the jumper must generate the appropriate forward and vertical linear momentum during the takeoff phase for their body to travel the correct distance to the target^16,18^. For this reason the CoM takeoff velocity angle and magnitude must be well coordinated to generate a ballistic trajectory to reach the desired landing location.

**Fig. 2:**
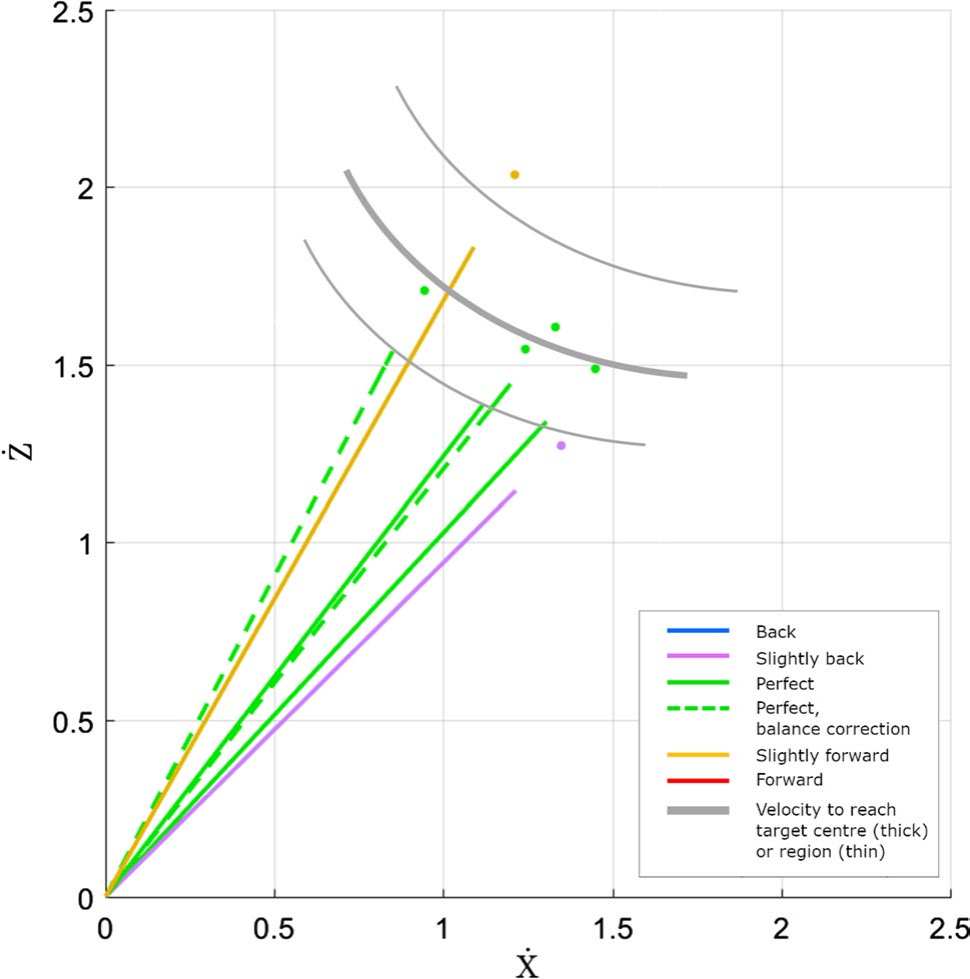
Example CoM takeoff velocity vectors for a set of six jumps from Participant 5, jumping to a target placed at 70% of their maximum jump distance. The line colours are the assigned jump grade and follow the convention outlined in Table 2. The dots denote the end of the vector for viewing clarity, where overlapping vector lines make it difficult to see where the vectors end. The thick grey curve identifies the hypothetical velocity required to reach the desired target distance of 0.8 m. Thin grey curves show an on-target region corresponding to an example target area, within which any takeoff velocity vector will result in a trajectory that lands in the target area.

Based on the relationship between takeoff velocity angle and magnitude, Fig. 2 shows an example set of CoM velocity vectors with the on-target zone approximated based on the data. Jumps that are coded as landing behind or ahead of the target correspond to takeoff angle and magnitude outside the on-target zone (Fig. 2, purple and yellow, respectively), while the remaining four jumps in the set were on-target, and were more closely clustered within the identified on-target zone. This behaviour is seen in the majority of the jumping experiment data.

### Foot placement and leg stiffness on landing

**Fig. 3:**
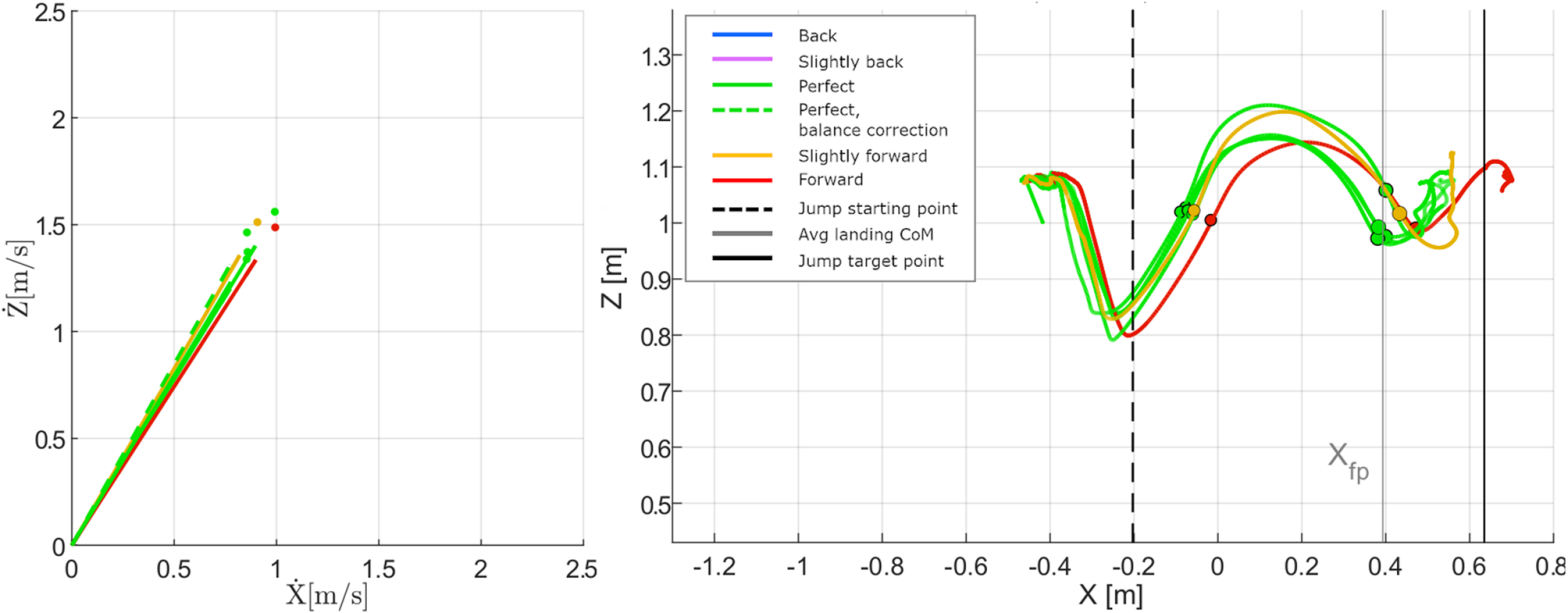
Six samples of takeoff velocities and corresponding CoM position trajectories from Participant 9, jumping to the 55% target. The line colours are the assigned jump grade and follow the convention outlined in Table 2. The dots (left) denote the end of the vector for viewing clarity where overlapping vector lines make it difficult to see where the vectors end. The circles (right) denote the CoM position when the feet left and regained floor contact. The dotted and solid vertical lines (right) show the location of the takeoff position and landing target. CoM trajectories behind, or forward of, the target distance exhibit early (legs extended), or late (legs partially collapsed) foot placement relative to on-target jumps. Average CoM forward position at landing is marked by the grey line at $X_{fp}$. Some trajectories (yellow, red) show poor foot placement timing that is likely the cause of failing a jump, even though takeoff velocity and vector are similar to successful jumps.

When jumping to a target, the landing position of the feet is specified prior to the jump (i.e. on the desired target). If the jumper’s takeoff velocity results in a trajectory that will not appropriately intersect the desired target, they can change their foot placement to recover from the inaccuracy of their initial takeoff velocity and still land on the target. If the trajectory is slightly short of the target, the jumper can collapse their legs while in the flight phase and use a later foot placement in order to travel the extra distance required to make it to the target. Alternatively, if the jumper’s trajectory will send them over the target, they can try to extend their legs and contact the ground sooner to prevent their CoM travelling too far over the target and stop their forward momentum in time. Incorrect foot placement can also be the cause of an unsuccessful jump, even if the initial trajectory is on target (Fig. 3).

Even after placing their feet on the ground, a jumper can affect how far forward their CoM travels by controlling their leg stiffness. Note that in this context, “leg stiffness” refers to the co-contraction of muscles around the hip, knee and ankle joints, which can control the mechanical impedance of the leg as well as the force produced by the foot on the ground^39^. Keeping the legs extended and stiff after foot contact causes a higher and faster impact, and quickly stops the forward momentum of the body. Alternatively, if the legs are allowed to collapse during landing, using greater flexion at the hips and knees, momentum absorption will occur over a longer period of time, and the CoM will travel farther forward during the landing phase.

If a jumper’s foot placement pose is not ideal at the moment of ground contact, the CoM can still be guided to a static position over the jumper’s support polygon at landing by controlling their leg stiffness after the feet touch the ground. As with foot placement, improper control of leg stiffness can also be the cause of an inaccurate jump. Two jumps can have similar CoM trajectory and foot placement, but if one jump has high leg stiffness, their CoM forward velocity will slow down quicker and end up short of the target.

### Motor learning

By comparing the jump trajectory data between the first and second sets of jumps to each target, motor learning effects of the participant can be observed. Table 2 shows that participants landed perfectly on target more often in the second set of jumps (66% of all jumps denoted as Set 2) compared to the first set (56% of all jumps denoted as Set 1) while decreasing in nearly all other categories of imperfect jumps. By assigning perfect jumps a value of 1, and 0 to all other types of jumps, the difference in grades between the two sets was found to be statically significant via the repeated measures analysis of variance (RM ANOVA) where the jump set and distance were the with-in subject variables ($F(1,19)=675.81,\,p<0.01$). The takeoff velocity was generally more consistent in participants second sets of jumps to each target, suggesting improved control for jumping to a particular distance after multiple repetitions of the movement.

Changes in foot placement pose between the first and second sets of jumps were less consistent. Some participants had noticeably tighter clustering of their foot placement in their second jump set suggesting these participants started controlling their foot placement more accurately the more they jumped (Fig. S6 in the Supplementary Materials S1). Most participants did not have a noticeable difference in foot placement clustering between their jump sets.

### Novice vs. expert technique

Expert jumpers generally controlled their foot placement pose and leg stiffness more effectively than novices. Even when some experts had less consistent takeoff velocities than novices, their overall jump success rate was higher because of their high level of foot placement control. This control allows expert jumpers to properly adapt their landing motions to counter inaccuracies in their takeoff velocity. Expert jumpers are more adaptable to varying jump conditions, hence their jumping success is more robust. This suggests that takeoff objectives are similar between novices and experts, experts’ performance differs from novices during landing. This is mirrored by a prior jumping study by McKinley^22^, where expert jumpers were observed to adjust for different landing conditions while novices did not, leading to more successful landings in the expert group in their analysis.

To analyse the differences between novice and expert jumpers, the following 2 metrics were statistically analysed using RM ANOVA, with the jump set and distance as the with-in subject variables and expertise as a between-subject variable: (1) variance of the CoM velocity vector^18^ at the point of takeoff, and (2) variance of the CoM velocity vector at the point of landing. These metrics were calculated by computing the variance over each jump set and distance for each participant. While the takeoff angle variance was not found to be statistically significant ($F(1,19)=2.60,\,p=0.12$), the landing angle variance was significant with respect to the participant’s expertise level ($F(1,19)=5.60,\,p=0.02$), which corroborates McKinley^22^’s findings.

## Inverse optimal control analysis

The IOC approach described in the Sect. 3 was then performed to identify the changing cost weights over the course of the jump. This analysis shows that all jumpers show an overall similar pattern, favouring CoM cost functions during takeoff, distance to target during flight, and CoM again during landing. Cluster analysis show four different jumping styles can be observed, where participants favoured a controlled takeoff (high CoM priority), rigid landing (did not bend legs on landing), explosive takeoff (high momentum on takeoff), or were hesitant jumpers (high task-space priority).

**Fig. 4:**
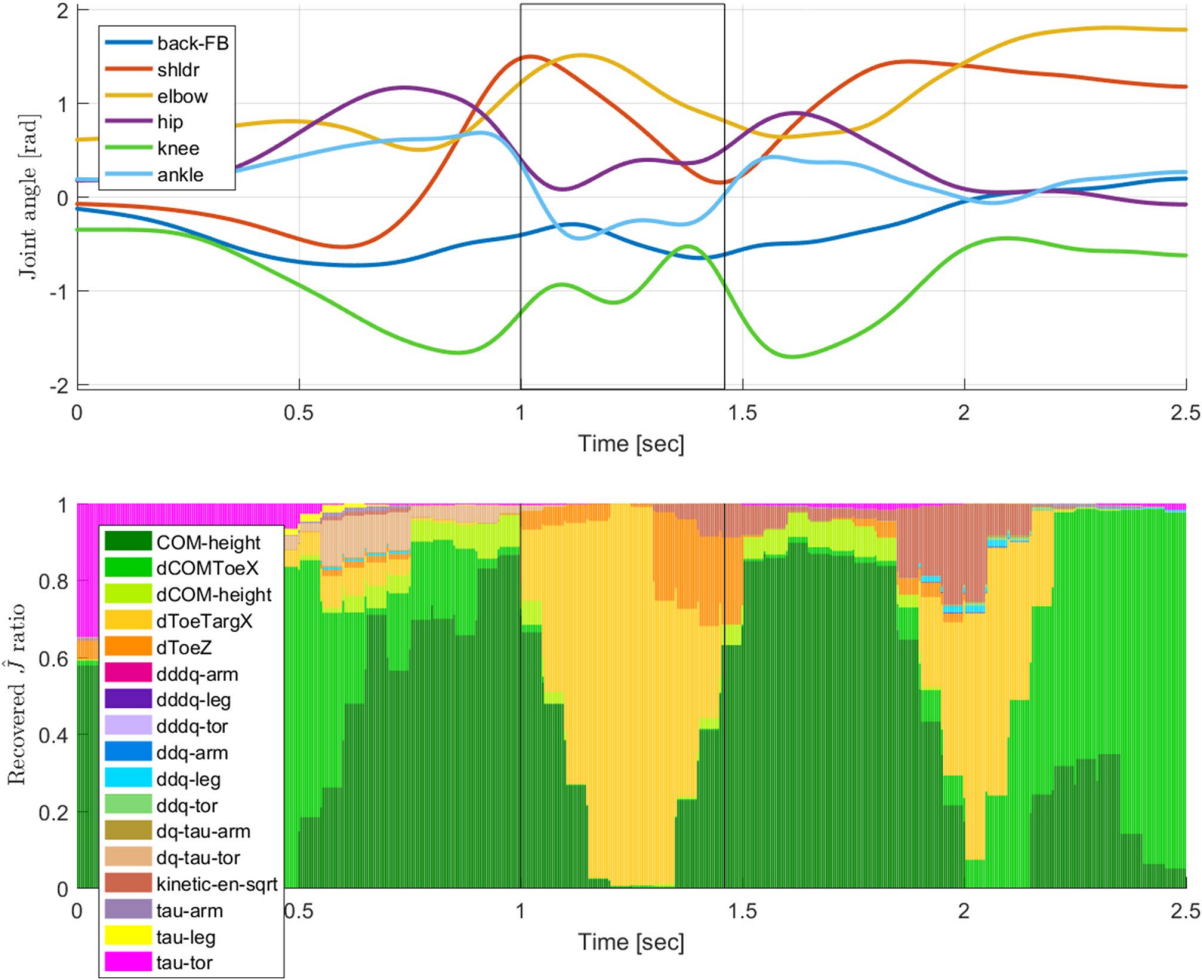
Example joint trajectories for a jump to a target, showing 6 of the 14 total DoFs (top), as well as the corresponding recovered IOC weights (bottom). Each frame consists of normalised recovered cost term weights, identifying the prioritised motor control tasks of the jumper throughout the movement. Takeoff and landing frames are marked with vertical black lines at 1.0 and 1.45 s, respectively. Note that this figure shows the weights of a single trajectory, while Figs. 5, 6, and 7 show averaged weights over several trajectories. The discretisation observed here is due to the window stride size.

### IOC cost term weight trajectory

**Fig. 5:**
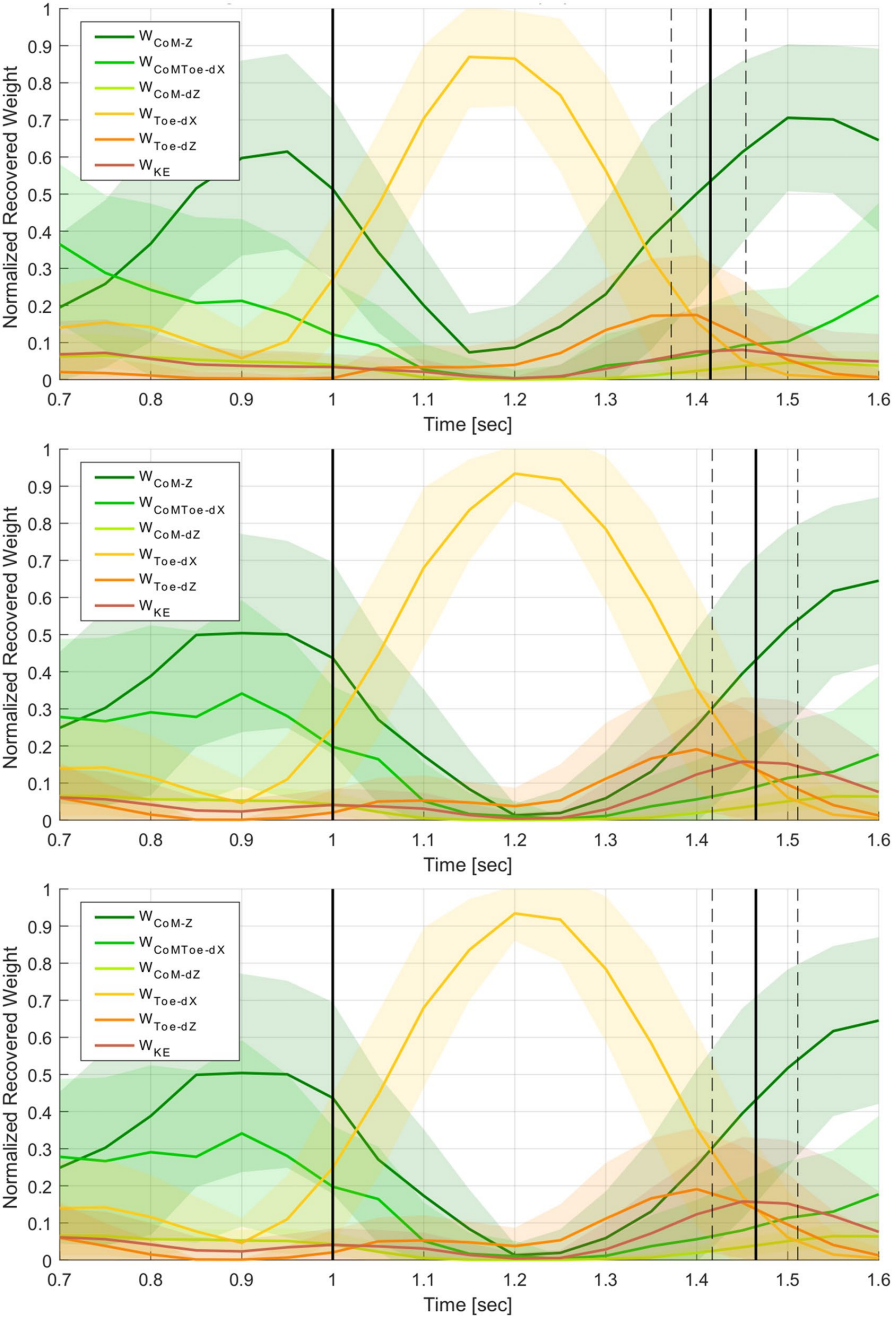
Comparison of mean weight trajectories for short 55% (top), medium 70% (middle), and long 85% (bottom) target distances, over all the participants, jump sets, and jump grades The takeoff and landing frames are marked with vertical black lines. Differences are seen in the $w_{CoM-Z}$, $w_{CoMToe-dX}$ and $w_{KE}$ trajectories.

Figure 5 shows the mean and standard deviations of the weight trajectories for each jump target over all participants and jumps. The cost term weight trajectories of all jumps contained common patterns. The most common patterns are: higher $w_{CoM-Z}$ and $w_{CoMToe-dX}$ during takeoff; mostly $w_{Toe-dX}$ during flight with $w_{Toe-dZ}$ and/or $w_{KE}$ just prior to landing; high $w_{CoM-Z}$, and sometimes a $w_{KE}$ peak, after landing.

Comparing the mean and standard deviation of weight trajectories between the targets, the most obvious difference is a shift of the landing weights further in time, due to the increased flight time required for longer target distances. $w_{CoM-Z}$ was found to differ significantly using RM ANOVA with the jump set and distance as the with-in subject variables at both takeoff ($F(2,38)=4.26,\,p=0.02$) and at landing ($F(2,38)=18.78,\,p<0.01$) over different jump distances. The changing $w_{CoM-Z}$ suggests that jumpers use a higher arcing trajectory for shorter jump distances, and a trajectory lower to the ground for longer jump distances. As $w_{CoM-Z}$ decreases over longer jumps, $w_{CoMToe-dX}$ was found to increase correspondingly ($F(2,38)=5.39,\,p<0.01$). $w_{KE}$ was also found to differ significantly between different jump distances, especially during the landing phase ($F(2,38)=14.89,\,p<0.01$). This is probably the result of generating more CoM momentum to reach the longer distance target, and consequently having to absorb more impact at landing (Fig. 4).

**Fig. 6:**
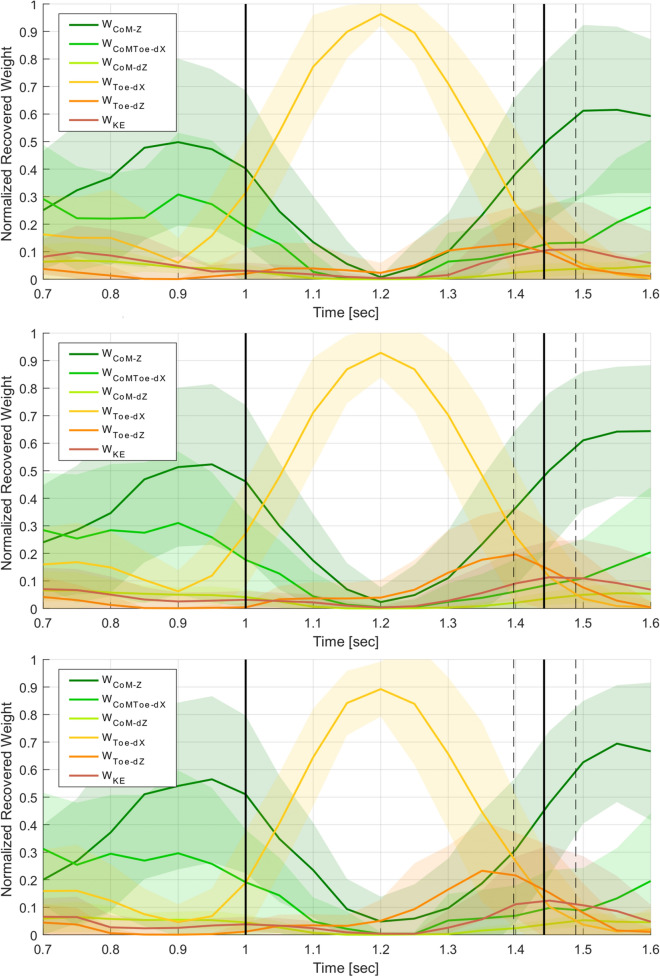
Mean weight trajectories for jumps to the medium distance target, comparing jumps graded as short of the target (top, grade=B,SB), on-target (middle, grade=P,P*), and overshooting the target (bottom, grade=SF,F). The takeoff and landing frames are marked with vertical black lines. Differences are seen in the $w_{CoM-Z}$ and $w_{Toe-dZ}$ trajectories.

Next, the mean weight trajectories were compared based on jump grading, for each target separately. Figure 6 shows mean weight trajectories for all medium target distance jumps, between jumps short of the target (graded B or SB), jumps on-target (P or P*), and jumps exceeding the target distance (SF or F). The primary weight trajectory differences relative to jump grade are observed in $w_{CoM-Z}$ and $w_{Toe-dZ}$. $w_{CoM-Z}$ in jumps short of the landing target are larger, but are smaller in overshot jumps. $w_{Toe-dZ}$ are lower in jumps short of the target but higher in jumps past the target. Using logistic regression to fit the scores relative to the cost weights, $w_{CoM-Z}$ at takeoff was found to be statistically significant when compared to perfect jumps if the the jump exceeded target ($T(1502)=2.06,\,p=0.03$), but not if the jump was short of the target ($T(1502)=0.55,\,p=0.58$). Conversely, marginal significance was found for $w_{Toe-dZ}$ at landing on jumps short of the target ($T(1502)=1.93,\,p=0.05$) but not if the jump overshot ($T(1502)=1.02,\,p=0.30$).

Mean trajectories of all on-target jumps have $w_{CoM-Z}$ and $w_{Toe-dZ}$ trajectories between the aforementioned extrema. This suggests a gradient in the weight trajectory changes between too short and too far over the target distance, with a middle range of motor control trajectory behaviour that results in an on-target jump. These observations are made based on motor control patterns for jumps to the medium distance target, but similar observations are seen for the short and long target distances. Similar gradient behaviour was observed in Sect. 4, based on the CoM trajectory data. CoM takeoff velocity vectors needed to be within the “on-target zone” to appropriately launch the body towards the target; the change in $w_{CoM-Z}$ discussed above could be a result of this change in takeoff velocity. Also, foot placement needed to occur when the CoM was at a specific horizontal distance behind the target, which may relate to the peak height of $w_{Toe-dZ}$ prior to landing.

**Fig. 7:**
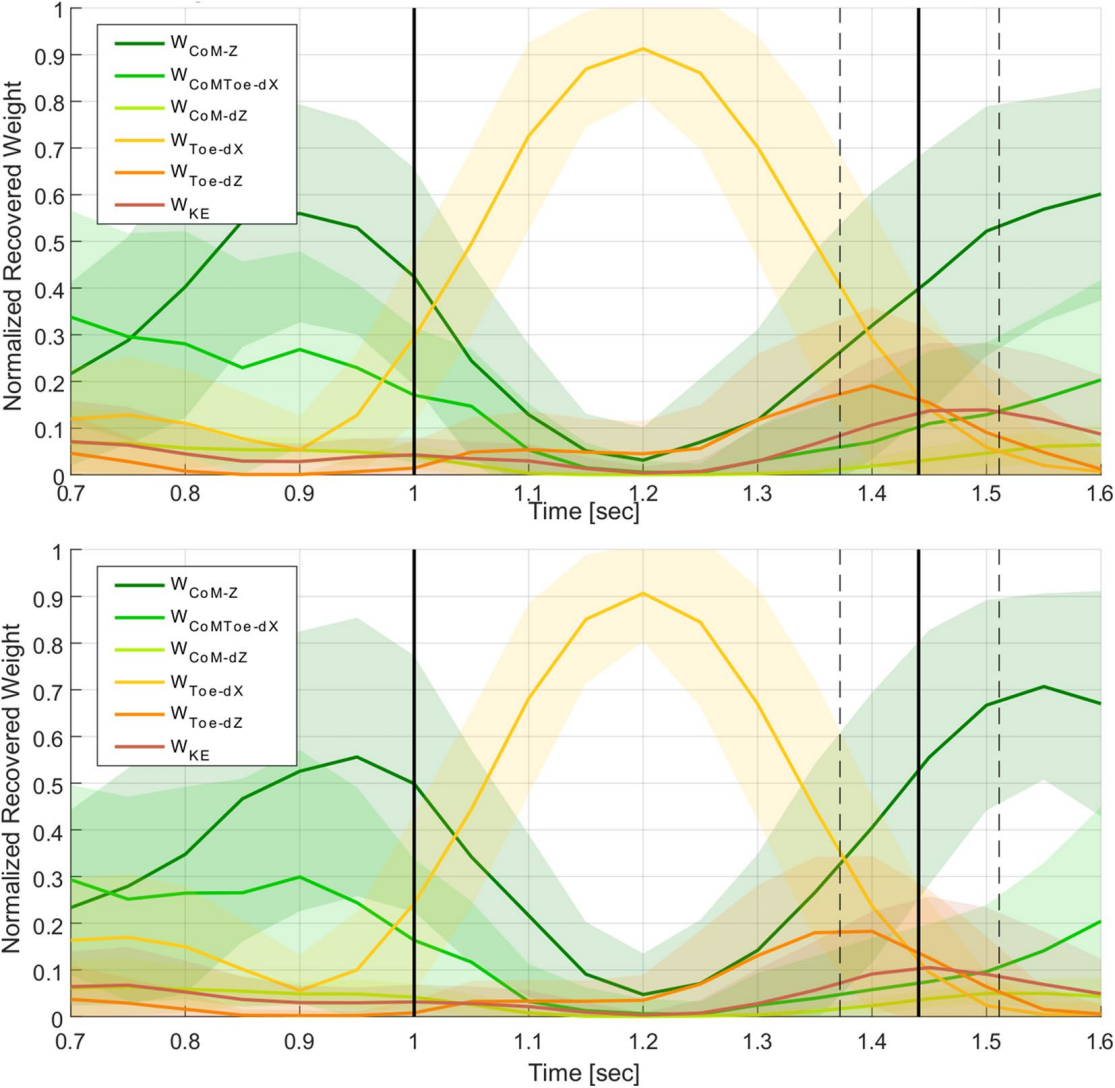
Comparison of mean weight trajectories for the first set of jumps (top) and novice (bottom) participants. The takeoff and landing frames are marked with vertical black lines. Differences are seen in the $w_{CoM-Z}$ and $w_{CoMToe-dX}$ trajectories.

Comparing expert and novice jumpers (Fig. 7), the primary difference between the mean weight trajectory plots is in $w_{CoM-Z}$. The expert jumps show marginal difference in $w_{CoM-Z}$ during the landing phase ($F(1,19)=2.96,\,p=0.10$) compared to the novice jumps. This trend suggests that the motor control patterns of novice jumpers change to resemble the average pattern seen in expert jumpers as novices practice more jumps. Expert jumpers by definition had higher jump success rates than novice jumpers, and the success rates of almost all participants were higher in their second jump set as compared to their first. Similar trends are seen for the short and long target distances. Combining the observations from the novice/expert and motor learning weight trajectory comparisons indicates that a motor control pattern that puts more priority to $w_{CoM-Z}$, results in a higher average jump success rate.

In general, $w_{CoM-Z}$ and $w_{CoMToe-dX}$ during the takeoff phase, and $w_{Toe-dZ}$ and $w_{KE}$ near or at landing, are the cost weights that vary the most relative to jump grading, target distance, jumper performance and motor learning.

### IOC weight trajectory clustering

The mean weight trajectory comparisons in the previous section were made by manually separating the jumping data into specific groups designed to identify motor control differences related to specific jump features. To determine if the recovered weights can be used to automatically group together similar motor control patterns, unsupervised clustering was performed on the IOC weight trajectory data for each jump.

The kmeans++ algorithm^35^ was used to cluster the recovered cost term weight trajectories, where weight trajectories from one jump formed one observation. To preserve temporal alignment of trajectories, jumps to each target distance were clustered separately (as for the mean weight trajectory comparisons from the previous section). Clustering data for jumps to the middle distance target are presented in this section, and similar results were found for the short and long distance target jumps. The elbow method was used to determine the number of clusters to use^40^, resulting in four clusters.

Jumps performed by a single jumper tended to be clustered in the same group. When comparing the jumpers in each group to their respective motion capture and trajectory data, the cluster groups seem to be indicative of “jumping style”, the coordination technique a jumper uses to perform the jump, and are not directly related to target distance, jump success rate (i.e. novice or expert designation), or motor learning.

Based on the video and kinematic visualisation of the data clustered in each cluster, the four jumping styles were characterised as: (A) controlled takeoff and landing, (B) stiff legged landing, (C) explosive takeoff, and (D) hesitant landing. Three of the participants (ID 3, 13, and 19) were not included into a single group because their jumps were categorised into 3 or all 4 of the jump style groups, whereas most of the jumps from other participants were sorted into one predominant group. Figures outlining the different jumping styles can be found in the Supplementary Materials (Figs. S7, S8, S9, and S10).

To compare the motor control strategies of each group, the mean and standard deviation of the IOC weight trajectories of all participants in each group were compared.

Group A jumpers exhibit controlled takeoff and landing, characterised by a very high $w_{CoM-Z}$ just prior to takeoff, as well as high $w_{Toe-dZ}$ before landing. Additionally, the middle of the flight phase exhibits almost 100% weight from $w_{Toe-dX}$, more than any other group.

Group B jumpers exhibit stiff legged landing. These jumpers typically left the ground with less vertical momentum, moved through the flight phase with extended legs, and kept their legs stiff and extended during the landing phase (rather than collapse their legs and lower their CoM, as jumpers in other groups did). Group B is characterised by a lower $w_{CoM-Z}$ and higher $w_{CoMToe-dX}$ trajectory than all other groups during takeoff. Also, at landing the average $w_{KE}$ is higher than normal.

Group C jumpers exhibit explosive takeoff. As can be inferred from the group name, jumpers of this style generated more momentum, jumped to farther target distances than those in other groups (based on their maximum distance calibration jumps), and brought their CoM low to the ground during the landing phase by collapsing their legs more than average. Group C is characterised by a high $w_{CoM-Z}$ during landing and a very low $w_{CoMToe-dX}$ during takeoff. This is the only group with a peak in their $w_{Toe-dZ}$ trajectory just after takeoff, and with the highest $w_{Toe-dZ}$ peak at landing of any group. These jumpers also have a high $w_{KE}$ peak that is quite late after landing, whereas the average jumper has a peak $w_{KE}$ at or just after landing.

Group D jumpers exhibit hesitant landing. Group D is characterised by a pre-landing peak in $w_{Toe-dZ}$ earlier than usual. These jumpers also had the highest standard deviation in their jump trajectories of any group, meaning they were the least consistent in their motor control behaviour. Jumpers in this group had lower average takeoff velocity magnitudes than other groups, but no other features were found to distinguish this group’s kinematic trajectory data from those of other groups.

It is important to note that all jump style groups show weight trajectories with smaller standard deviations than the previous “manually grouped” sets of jumps (with respect to target distance, jump grade, jumper success rate and motor learning), even when compared to the higher standard deviation of group D. The smaller standard deviation within these groups is expected, since the purpose of unsupervised clustering is to group together data points that are most similar to each other. The clustering comparisons also show that there are several possible variants to the general motor control pattern that people use when jumping to a target.

To determine if any of the identified differences are statistically significant, RM ANOVA was performed with jump set as the within-subject variable. Differences between clusters in $w_{CoM-Z}$ on takeoff ($F(3,50)=6.74,\,p<0.01$) and landing ($F(3,50)=14.51,\,p<0.01$), maximum $w_{CoMToe-dX}$ over the trajectory ($F(3,50)=2.96,\,p=0.04$), and $w_{Toe-dZ}$ on landing ($F(3,50)=37.02,\,p<0.01$) were found to be statistically significant. Differences between clusters for $w_{KE}$ on landing ($F(3,50)=2.42,\,p=0.07$) were found to be marginally significant.

## Conclusion

In this paper, the movement of jumping to a target was investigated to develop and test a framework for the analysis of dynamic human motions. This framework focuses on movement for task optimisation, where a metric of task success can be clearly defined. The motion capture kinematic data of jumping movements performed by 22 participants were captured and graded based on distance to jump target.

These kinematic trajectories were analysed to find trends between trajectory features and jump success. The two trends most influential to jump success were the CoM takeoff velocity, which guided the body along an appropriate ballistic trajectory to the desired target, and the foot placement pose at landing, which controlled CoM momentum absorption, landing balance, and corrected for inaccuracies in CoM takeoff velocity.

An IOC sliding window approach was used to examine motor control behaviour of the experimental jumping data. The control tasks which varied most relative to these jump characteristics were $w_{CoM-Z}$ and $w_{CoM-dX}$ during the takeoff phase, and $w_{Toe-dZ}$ and $w_{KE}$ at and after landing. Clear changes in the optimised control tasks were observed relative to different target distances, jump grade, and average jumping success rate. Unsupervised clustering was used to identify motor control patterns corresponding to four distinct jumping styles observed in experiment participants, demonstrating that multiple control strategies can be used to successfully jump to a target.

As novice jumpers gained more practice, their landing accuracy improved due to a more consistent takeoff trajectory, while foot placement did not noticeably change.

For future work, additional data collection involving more challenging jump conditions can augment the analysis of unsuccessful jumps, such as using a more narrow landing target, jumping in different environments, or varying the style of jumps. The collected dataset can also be used to investigate alternative jumping models and control hypotheses.

## Supplementary information

Supplementary file1 (ZIP 796 kb)

## Data Availability

The motion capture marker position data and the reconstructed joint angle estimates are provided. Please see the Supplementary Dataset Descriptors for additional details.
