# Supplementary material for: Inverse optimal control with time-varying objectives: application to human jumping movement analysis
Source: Sci Rep. 2020 Jul 7;10:11174. doi: 10.1038/s41598-020-67901-x (PMC7341860; doi:10.1038/s41598-020-67901-x)
Supplement: Supplementary file 1 — Supplementary file1 (ZIP 796 kb) [file 41598_2020_67901_MOESM1_ESM.zip › Supplementary/tex_appendix.pdf]

Inverse Optimal Control with Time-Varying Objectives: Application to Human Jumping Movement Analysis (Supplementary Materials)

Kevin Westermann[1], Jonathan Feng-Shun Lin[1, *], and Dana Kulić[1, 2]

[1]University of Waterloo, Waterloo, Canada
[2]Monash University, Melbourne, Australia
[*]Corresponding Author (email: jf2lin@uwaterloo.ca)

ABSTRACT

This document serves as the Supplementary Material to the manuscript 'Inverse Optimal Control with Time-Varying Objectives: Application to Human Jumping Movement Analysis'. It provides in-depth background information on the data collection methodology and how to utilized the included jumping dataset. It also contains several figures and tables to provide background information and to expands on the results outlined in the main manuscript.

Supplementary Dataset Descriptors

The attached dataset was collected and analysed for this manuscript. The following section includes participant demographic data, kinematic model parameters, motion capture and joint trajectories, and temporal trajectory alignment information. Entities denoted in this `font` denote a reference to the attached files, or a variable within the files.

Data Collection

The methods used to create this dataset can be separated into four steps: (1) data collection and pre-processing, (2) developing the kinematic model, (3) pose estimation, and (4) temporal data alignment. The flow chart below (Figure S1) illustrates the dataflow and corresponding dataset files.

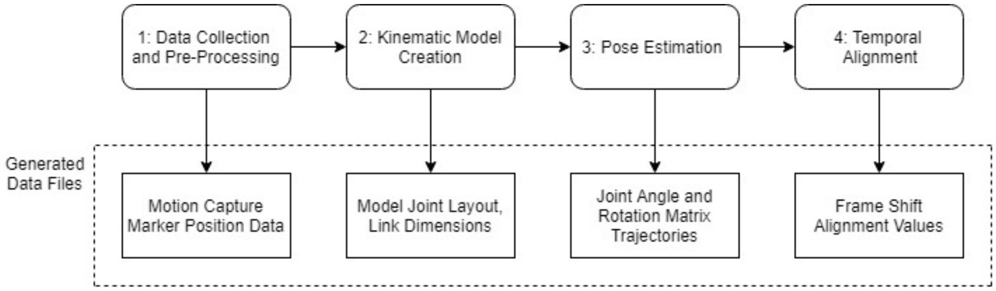

Figure S1. Dataflow chart for the jumping dataset.

Motion Capture System and Participant Setup

The motion capture equipment used in the study was an 8 Eagle-camera Motion Analysis infrared camera system. A total of 22 participants were recruited, 9 females and 13 males, between the ages of 19 and 34. Any participants with any serious injuries in the previous 6 months were excluded from the study. Each participant filled out a demographic information and eligibility form before the data collection (see `participant_demographic_data.csv`). This experiment was reviewed by the University of Waterloo Research Ethics Board, and informed consent was obtained from all participants. Note that motion capture data from Participant ID 01 contained too many marker occlusions and mislabelling errors, and so this dataset includes data from participant IDs 02 to 22.

Each participant was outfitted with 30 reflective markers at the body locations shown in Figure S2. 3D global marker positions were recorded at 200Hz. Markers were attached with hypoallergenic tape directly to the skin where possible or on top

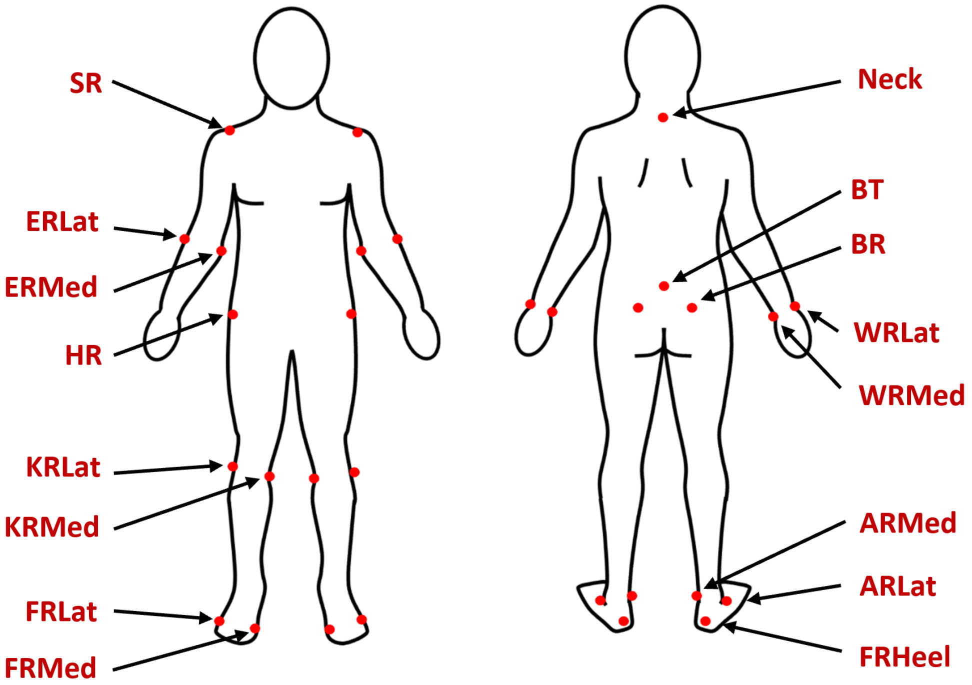

Figure S2. Motion capture marker positioning diagram. 30 markers were used to track the position of the body. Only right side markers are labelled in the diagram, while corresponding left side marker names replace the second “R” character with “L”.

of tight-fitting clothing. All markers were labelled based on their attachment point on the body. Table S1 lists the marker labels, full names, and detailed attachment points.

Table S1. Motion capture marker labelling, placement and attachment location.

Num	Label	Name	Body Placement	Frame Attachment
1	Neck	Neck	Back of neck at C7	spine @ upperBack
2	SL	Shoulder Left	Acromion process	spine2shldr_L @ shoulder_L
3	SR	Shoulder Right	Acromion process	spine2shldr_R @ shoulder_R
4	HL	Hip Left	Anterior superior iliac spine (ASIS)	base (after world2base)
5	HR	Hip Right	ASIS	base (after world2base)
6	BT	Back Top	Back of waistband, approximately L5	base (after world2base)
7	BL	Back Left	Posterior superior iliac spine (PSIS)	base (after world2base)
8	BR	Back Right	PSIS	base (after world2base)
9	ELLat	Elbow Left, Lateral	Humerus lateral epicondyle	uparm_L @ elbow_L
10	ELMed	Elbow Left, Medial	Humerus medial epicondyle	uparm_L @ elbow_L
11	WLLat	Wrist Left, Lateral	Styloid process, ulna	forearm_L @ wrist_L
12	WLMed	Wrist Left, Medial	Styloid process, radius	forearm_L @ wrist_L
13	ERLat	Elbow Right, Lateral	Humerus lateral epicondyle	uparm_R @ elbow_R
14	ERMed	Elbow Right, Medial	Humerus medial epicondyle	uparm_R @ elbow_R
15	WRLat	Wrist Right, Lateral	Styloid process, ulna	forearm_R @ wrist_R
16	WRMed	Wrist Right, Medial	Styloid process, radius	forearm_R @ wrist_R
17	KLLat	Knee Left, Lateral	Femur lateral epicondyle	thigh_L @ knee_L
18	KLMed	Knee Left, Medial	Femur medial epicondyle	thigh_L @ knee_L
19	ALLat	Ankle Left, Lateral	Fibula apex of lateral malleolus	shin_L @ ankle_L
20	ALMed	Ankle Left, Medial	Tibia apex of medial malleolus	shin_L @ ankle_L
21	FLLat	Foot Left, Lateral	5th metatarsus head (base of 5th toe)	foot_L @ toe_L
22	FLMed	Foot Left, Medial	1st metatarsus head (base of big toe)	foot_L @ toe_L
23	FLHeel	Foot Left, Heel	Posterior Surface of Calcaneus	foot_L @ toe_L
24	KRLat	Knee Right, Lateral	Femur lateral epicondyle	thigh_R @ knee_R
25	KRMed	Knee Right, Medial	Femur medial epicondyle	thigh_R @ knee_R
26	ARLat	Ankle Right, Lateral	Fibula apex of lateral malleolus	shin_R @ ankle_R
27	ARMed	Ankle Right, Medial	Tibia apex of medial malleolus	shin_R @ ankle_R
28	FRLat	Foot Right, Lateral	5th metatarsus head (base of 5th toe)	foot_R @ toe_R
29	FRMed	Foot Right, Medial	1st metatarsus head (base of big toe)	foot_R @ toe_R
30	FRHeel	Foot Right, Heel	Posterior Surface of Calcaneus	foot_R @ toe_R

Experiment Protocol (ordering)

Each participant first completed a warm-up routine. All jumping motions in the study were standing broad jumps to a target, starting with both feet on the ground, jumping with both feet leaving the ground at approximately the same time (takeoff), and then landing with both feet contacting the ground at approximately the same time (Figure 1).

At the beginning of each jump recording the participant stood in a calibration pose for two seconds, and then dropped their arms to their sides and stood upright for two seconds. The jumping action was then completed, and after landing and stabilisation the participant entered the calibration pose once more before the recording was stopped. The calibration pose was either the T-pose (straight arms raised to the side at 90 degrees) or the L-pose (arms raised forward with elbows bent at 90 degrees, humerus parallel to ground and forearm vertical with hand up). The T-pose was originally used since it is a standard for pose estimation calibration, however, the kinematic model we used contained a singularity at the shoulder when the arms were fully abducted and so we changed to using the L-pose.

Each participant first completed a set of three jumps for maximum distance. The jumps were measured from the takeoff line to the heel of the foot farthest back in the landing position, then the average of the measured distances formed the participant's first calibration distance, `CAL_1`. This distance was then used to compute the three *target jump* distances.

Next, the target landing platform was anchored to the floor. This platform is a 3/8" thick foam flooring mat, measuring 8" wide and 24" long, with the long dimension placed perpendicular to the jump direction. Three takeoff lines were marked on the floor at distances of 55%, 70%, and 85% of the `CAL_1` distance, measured from the centre of the target platform. Participants completed a set of 6 jumps to each target, starting from the shortest (55%) to longest (85%) distance. For each jump, the takeoff line was recorded with three markers on the line, and the target platform was recorded with four markers, one at each corner.

The protocol described above was then repeated a second time. A second set of three maximum distance jumps were completed and `CAL_2` was calculated. If `CAL_2` was less than `CAL_1`, the second set target distances remained scaled based on `CAL_1`. Otherwise, three new target distances were calculated as 55%, 70%, and 85% of `CAL_2`, and sets of 6 jumps were completed to each of these new target distances. This resulted in 36 total jumps completed by each participant. The flow chart below (Figure S3) illustrates the experiment protocol steps.

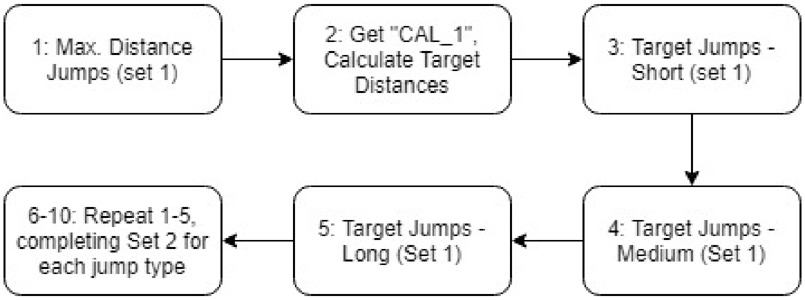

Figure S3. Protocol flow chart.

Participants 02, 14, 17 and 20 were able to jump far enough (based on their jumping ability or relative height) that the capture volume of the motion capture system was not large enough to accurately record the takeoff and landing motions. In these cases the 85% jump distance (third target) was made less than the actual 0.85· `CAL_#` value in order to remain within the capture volume of the motion capture system.

Experiment Questionnaire

Before the experiment and after each set of jumps each participant answered questions about their current fatigue level. Additional questions dealing with stress levels, jumping improvement and performance were asked before, in the middle of, and after the data collection. All questions were answered with a number between 1 and 10, with 10 representing the highest fatigue/stress rating, and the most positive improvement/performance rating. Table S2 presents the order and detailed description of all questions asked throughout the data collection.

Data Post-processing

The raw marker position data contained minor occlusions or incorrectly labelled markers. All jumping data of all participants were manually post-processed in the Motion Analysis system's native software Cortex. After fixing all labelling and occlusion errors, marker position trajectories were smoothed at 6 Hz.

Each of the 36 jumps performed by each participant has two motion capture data files associated with it, one for the 30 body markers and a second one for the 7 markers showing the position of the takeoff line and target platform. The marker positions in the second file can be used to calculate the distance of each jump, which remained constant for each set of 6 jumps. See the Data Records section for detailed information on the motion capture data file format and naming convention.

Table S2. List of experiment questions. (B) or (A) indicate that a question is asked before (B) or after (A) each target (T) or set (S). Question short form names correspond to the header row of the `participant_questionnaire.csv` data file.

Num	When is Question Asked?	Full Question	Short Form Name
1	B max. distance	What is your current level of fatigue?	Pre Exp F
2		What is your current level of stress?	Pre Exp Stress
3	A max. S 1	What is your current level of fatigue?	Max_1 F
4	A T 55 S 1	What is your current level of fatigue?	Target_1_1 F
5	A T 70 S 1	What is your current level of fatigue?	Target_1_2 F
6	A T 85 S 1	What is your current level of fatigue?	Target_1_3 F
7	A max. S 2	What is your current level of fatigue?	Max_2 F
8		What is your current level of stress?	Mid Exp Stress
9		How well do you feel you have improved your jumping ability so far?	Mid Exp Improve
10		What is your perception of your performance so far?	Mid Exp Perform
11	A T 70 S 2	What is your current level of fatigue?	Target_2_1 F
12	A T 85 S 2	What is your current level of fatigue?	Target_2_2 F
13	A T 85 S 2	What is your current level of fatigue?	Target_2_3 F
14		What is your current level of stress?	Post Exp Stress
15		How well do you feel you have improved your jumping ability so far?	Post Exp Improve
16		What is your perception of your performance so far?	Post Exp Perform

Kinematic Model

A kinematic model was developed to approximate the human skeleton for pose estimation (described in the next section). The model is formed with rigid links connected with prismatic and revolute joints, with a total of 15 links and 35 degrees of freedom. Sets of three orthogonal revolute joints represent the shoulders and hips. One prismatic joint was added to each shoulder joint, variable along the frontal axis of the body, to better approximate scapula movement. Figure S4 shows a labelled diagram of the links and frame/joint locations of the kinematic model.

In the *zero pose* (Figure S4) the spine, upper arm, forearm, thigh and shin links have only a vertical offset component from their origin frame, while the *spine2shldr*, *base2hip*, and foot links have 3D offsets. All joints are at zero degrees in the zero pose. Table S3 lists all joints in the kinematic model and where they are located, in the order they are listed in the joint angle data files (described further in the Data Records section). The positive angle direction of each joint is in the same direction as the joint name (ie. sagittal plane knee joint is named `knee_jExtension`, so increasing angles mean the leg is straightening and decreasing angles refer to the knee flexing).

The kinematic model link offsets were calculated for each participant based on the motion capture marker positions recorded during the calibration pose. The calibration window used for all kinematic model calculations was over one full second, from frames 251 to 450. This window was optimised to reduce the marker position error over all joint angle data. Elbow, wrist, knee and ankle joint centres were assumed to be located directly between markers placed on either side of these joints. The model base frame and hip joint centres were calculated based on Harrington's anthropometric equations[1], using the markers on the front (ASIS) and rear (PSIS) of the pelvis, and the leg length.

The shoulder joint centres of the model were assumed to be directly under each shoulder marker (under the acromion process) when in the zero pose, by a constant distance *S2sc* that was estimated to the nearest 5 mm during data collection.

Model link dimensions were first calculated based on the calibration window of each jump recording, and then were averaged to form the final kinematic model used for all jumps performed by the participant.

Markers were then rigidly attached to the model at specific locations. Table S1 includes the model links to which each marker was attached. Although the same model dimensions were used for all jumps performed by a participant, in order to consistently compare joint angles from different jumps, the markers were attached to the model based on the calibration pose of each individual jump. This prevented small changes in marker position during data collection, due to clothing movement, sweat, marker reattachment, etc., from affecting the accuracy of the joint angles calculated from pose estimation.

Table S3. Kinematic model joint names and locations. Joint names are ordered as they appear in joint trajectory data files.

Number	Name	Located At	Joint Colour in Fig S4
1	pjoint0	world2base	N/A
2	pjoint1		
3	pjoint2		
4	rjoint0		
5	rjoint1		
6	rjoint2		
7	back_jFB	base	blue
8	back_jAxial		blue
9	back_jLateral		blue
10	rshoulder_jprism	between upperback and rshoulder	N/A
11	rshoulder_jElevation	shoulder_R	orange
12	rshoulder_jAbduction		green
13	rshoulder_jExtRotation		blue
14	relbow_jFlexion	elbow_R	orange
15	relbow_jSupination		green
16	lshoulder_jprism	between upperback and lshoulder	N/A
17	lshoulder_jElevation	shoulder_L	orange
18	lshoulder_jAbduction		green
19	lshoulder_jExtRotation		blue
20	lelbow_jFlexion	elbow_L	orange
21	lelbow_jSupination		green
22	rhip_jFlexion	hip_R	orange
23	rhip_jAbduction		green
24	rhip_jExtRotation		blue
25	rknee_jExtension	knee_R	orange
26	rknee_jExtRotation		green
27	rankle_jDorsiflexion	ankle_R	orange
28	rankle_jPronation		green
29	lhip_jFlexion	hip_L	orange
30	lhip_jAbduction		green
31	lhip_jExtRotation		blue
32	lknee_jExtension	knee_L	orange
33	lknee_jExtRotation		green
34	lankle_jDorsiflexion	ankle_L	orange
35	lankle_jPronation		green

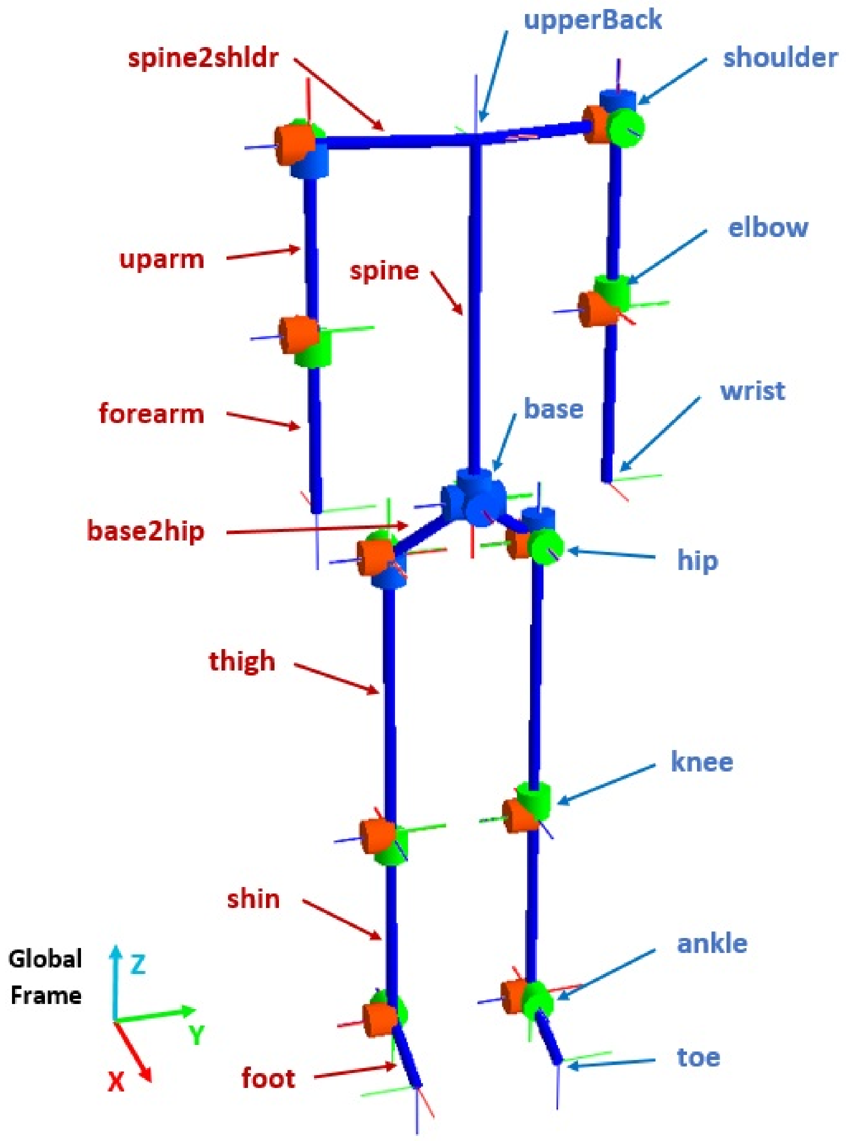

Figure S4. Kinematic model in the *zero pose*. Frame locations are labelled in blue and links in red. The coloured cylinders denote revolute joints, with the red, green, and blue colouring to denote the joint order in the cases on concentric joints. All frames/links with a left and right side have _L or _R appended to the name, respectively. The *world2base* offset is between the global frame and the base frame.

Pose Estimation

The marker position data was input to an extended Kalman Filter (EKF)[2] for estimating the pose of the participant at each frame of the jump recording, using the joint angles, velocities and accelerations of the kinematic model as the state.

The primary metric of pose estimation accuracy was the mean absolute error (MAE) between the ground truth marker positions from motion capture and the estimated positions of the markers based on the joint angle estimates computed from EKF.

$$\text{MAE} = \frac{\Sigma_{i=1}^{n}\sqrt{(x_{t,i}-x_{e,i})^2+(y_{t,i}-y_{e,i})^2+(z_{t,i}-z_{e,i})^2}}{n}$$

where $(x_t, y_t, z_t)_i$ is the true marker position, and $(x_e, y_e, z_e)_i$ is the estimated marker position at the i-th index of the summation variable. The position errors can be summed over all frames, all markers, or combinations of both (see the Technical Validation section for examples).

A constant acceleration model was used for the state update step. Observation noise was set to 0.01 (average marker MAE at or below 10 mm) and process noise was optimised to a value of 1.05 by minimising the average MAE from all markers, over all jumps and participants.

Pose Estimation Results

The results of the pose estimation consist of the initial *world2base* transform to locate and orient the kinematic model in the starting position, and the joint angle trajectories for moving the kinematic model through the jumping action. The positions of the markers attached to the model can be compared to the ground truth marker positions as a metric of pose estimation accuracy (please see the Technical Validation section).

Euler Angle Gimbal Lock

One issue with the joint angle results is the effects of entering the gimbal lock singularity, since spherical joints are represented as 3 orthogonal Euler angle joints in the kinematic model.

To accurately represent the rotations of the spherical joints without the representational singularity, 3D rotation matrix trajectories were generated for the shoulders and hips, in addition to the single revolute joint angle trajectories. Joint angle and rotation matrix trajectory data are described in the Data Records section.

Data Alignment

The trajectory data is not temporally aligned, since each participant does not jump at exactly the same time during each jump relative to when the recording starts. In order to more easily compare the trajectories, a temporal shifting algorithm was applied to the jump data. Trajectories were shifted relative to one another by integer frame values. The temporal alignment values can be applied to the joint trajectories, spherical joint rotation matrix trajectories, and the Cartesian marker position data.

The 12 jumps performed to each target distance were aligned to each other, since the temporal variation between trajectories to the same target were negligible (even after slight differences in the target distances based on `CAL_1` and `CAL_2`, as described in the Data Collection section. Jumps to different target distances varied too much to compare their trajectories directly, since flight times were significantly longer for farther jump distances, and jump technique sometimes changed for different target distances. The 12-th jump (6-th jump of the second set) to each target was used as the reference trajectory for the alignment algorithm, and the 11 other jumps to each target were shifted to match the reference trajectory. Only temporal shifting of the entire trajectory was used to align the jumps.

Rough Alignment

First, the data frames where the participant left the ground (*takeoff*) and landed on the target platform were determined. Takeoff frames were identified as the first frame where the average speed of the medial foot markers was greater than 0.2 m/s just before takeoff. Landing frames were identified as the first frame after takeoff when the average medial foot marker speed was less than 0.2 m/s. Then, a rough alignment was performed, aligning the landing frames of each group of 12 jumps to each target.

Fine Alignment

After rough alignment, the jumps were further aligned by minimising the sum of absolute error between the shifted signals and reference signals for hip flexion, knee extension, and ankle dorsiflexion joints (right and left). The resulting shift values for each of these 6 joints were then averaged to get the final shift value.

Only the hip flexion, knee extension and ankle dorsiflexion joints were used to align the joint trajectories because the other joint trajectories were much more variable across several jumps.

The final shift values to align each jump to the 12-th reference jump are available in the dataset, and are presented in the Data Records section. A positive shift value corresponds to shifting the trajectory earlier in time (i.e. removing frames from the front of the trajectory), while a negative shift value means shifting the trajectory later in time (i.e. adding placeholder frames to the front of the trajectory). This process is demonstrated in the sample code available with the dataset (see the Code Availability section).

Code Availability

Sample MATLAB scripts are included that demonstrate (1) loading participant demographic data, (2) loading and plotting Cartesian marker position data for a single jump, and (3) loading all 12 jump trajectories to a specific target and temporally aligning and plotting the trajectories. This code provides all basic operations to load, view and interpret the data.

To solve the inverse kinematics and generating the joint angle data, we used an implementation of EKF[2]. Full details on the EKF setup and parameters are explained in the Pose Estimation section.

Data Records

All data are provided in comma separated values (.csv) or tab-delimited text (.trc) formats.

Data files are organised into three sections: (1) demographic and questionnaire data for each participant, (2) motion capture marker position data, and (3) kinematic model and joint angle trajectory data. The joint angle trajectories also have associated data to temporally align the jumps of each set.

All data files and folders specific to one participant start with the naming convention P##, where ## corresponds to the participant number.

Table S4. Naming convention and location of all data files in the dataset. Data is the parent folder for the dataset. ## corresponds to the participant ID number.

File Name	File Location
Data/	participant_demographic_data.csv<br>participant_questionnaire.csv<br>model_joint_names.csv
Data/P##_Data/	P##_jump_grades.csv<br>P##_model_links.csv<br>P##_world2base.csv<br>P##_takeoff_land_frames.csv<br>P##_ shift_align_values.csv
Data/P##_Data/Motion Capture	P##_target_&&_&_&_clean-P##_template.trc<br>P##_target_&&_&_&_clean-start_land.trc
Data/P##_Data/Joint Angles	P##_JA_jump_$.csv<br>P##_JA_jump_$_R-shldr_L.csv<br>P##_JA_jump_$_R-shldr_R.csv<br>P##_JA_jump_$_R-hip_L.csv<br>P##_JA_jump_$_R-hip_R.csv

Table S4 lists all file types, naming convention, and where they are located. Detailed descriptions for using each file type are below. For all files including data pertaining to each of the 36 jumps performed by a participant, the jumps are organised in a specific order. The ordering hierarchy is first decided by the target jump distance (short = 55%, medium = 70%, then long = 85%), then by the set number to that target (1 or 2), and then by the jump number within that set (1 to 6). This is summarised in Table S5. Note that this is not the same order the jumps were completed in during data collection. Table S2 presents the full jump ordering.

While 22 total participants completed the data collection, the data from participant ID 01 contained too many marker occlusions and mislabelling errors to be useable. Therefore, data for participant ID 01 are not included in this dataset.

Demographic Data and Questionnaire Responses

participant_demographic_data.csv Located in the parent folder of the dataset, this file lists demographic and background data for all participants, including: age, gender [M/F], height [cm], weight [lbs], *S2sc* [cm], calibration pose [T/L], and self-evaluated physical activity level [hours per week]. *S2sc* represents the length from the shoulder marker to shoulder centre, and is an estimate of the vertical distance between each shoulder marker (on the acromion process) and the glenohumeral joint centre beneath it. This value is used when creating the kinematic model. The calibration pose is the pose each participant starts in during each jump recording. This can either be a T-pose (arms raised to 90 degrees, straight out to the side) or L-pose (arms raised forward with elbows at 90 degrees, humerus parallel to ground and forearm vertical up).

participant_questionnaire.csv Located in the parent folder of the dataset. Includes the answers to the experiment questionnaire for all participants. The experiment questionnaire is described in detail in the Data Collection section of this document. The header row of this file contains short-form labels of questions corresponding to each column of data.

Motion Capture Data

Motion capture data files are located in the P##_DataMotion Capture folder corresponding to each participant. These files are in a text format called .TRC, which is the marker position data output format used in the motion capture software. Each .trc file includes a header detailing parameters for the recording such as the number of markers, number of frames, framerate, etc.

Table S5. Jump ordering list for kinematic model and joint trajectory data. Note that this is not in the same order that the jumps were performed during data collection.

Jump Number	Jump in Set	Set to Target	Target
1	1	1	55 (short distance, first target)
2	2		
3	3		
⋮	⋮		
6	6		
7	1	2	
⋮	⋮		
12	6		
13	1	1	70 (middle distance, second target)
⋮	⋮		
18	6		
19	1	2	
⋮	⋮		
24	6		
25	1	1	85 (long distance, third target)
⋮	⋮		
30	6		
31	1	2	
⋮	⋮		
36	6		

After the header rows, the first two columns contain frame numbers and frame time [sec]. The third column onwards includes global X, Y and Z position data for each marker [mm], labelled in the header rows.

P##_target_&&_&_&_clean-¶##_template.trc Includes global marker position data of the 30 body markers on the participant. For each jump that each participant performs, there are two motion capture `.trc` files: one for the 30 body markers, and a second for the three start line and 4 landing platform markers, as described in the Data Collection section).

P##_target_&&_&_&_clean-start_land.trc Includes global marker position data of the three start line and 4 landing platform markers, as described in the Data Collection section. These data are almost constant during each set, but small disturbances occur to the landing platform markers when the participant lands. The `##` symbols are in place of the participant ID number associated with the data. The `&&_&_&` symbols are in place of the target distance (55, 70, or 85), set number (1 or 2), and jump number (between 1 and 6), in that order, associated with the motion capture recording. For example, file name `P03_target_70_2_1_clean-P03_template.trc` corresponds to the motion capture data of participant ID 3 making the first jump of their second set to the 70% distance target, which is the second target distance.

Performance, Kinematic Model and Joint Angle Data

These files are related to the jump performance by each participant, the kinematic model parameters used for the participant, the model joint angle results after pose estimation and jump alignment information.

P##_jump_grades.csv Located in each `P##_Data` folder, this file provides the assessment assigned to each jump by the experimenter, classifying the participant's ability to stick the landing on the target platform. Each of the 36 jumps in jump ordering are assigned one of 6 grades (B, SB, P, P*, SF, or F), as described in the Data Collection section.

P##_model_links.csv Located in each `P##_Data` folder, this file includes the dimensions of the kinematic model links used for each participant. Global frame offsets are presented for each of the 15 different links in the model while in the zero pose. The global frame is oriented with the +X axis pointing in the direction of the jump, and the +Z axis pointing upward.

P##_world2base.csv Located in each `P##_Data` folder, this file includes the homogeneous transformation matrices from the global frame to the base frame of the model. Matrices are presented for each jump of the participant in jump ordering, in a column-major vectorized format as shown below:

$$\begin{bmatrix} a & d & g & j \\ b & e & h & k \\ c & f & i & l \\ 0 & 0 & 0 & 1 \end{bmatrix} = \begin{bmatrix} a & b & c & 0 & d & e & f & 0 & g & h & i & 0 & j & k & l & 1 \end{bmatrix}$$

P##_takeoff_land_frames.csv Located in each `P##_Data` folder, this file includes the data frames where the participant left the ground (takeoff) and landed on the target platform. Takeoff frames are the first frame where the average speed of the medial foot markers was greater than 0.2 m/s just before take-off. Landing frames are the first frame after takeoff when the average medial foot marker speed was less than 0.2 m/s.

P##_shift_align_values.csv Located in each `P##_Data` folder, this file includes the number of frames required to temporally "shift" the data to align it with the other jumps to each target. A positive shift value corresponds to shifting the trajectory earlier in time (i.e. removing frames from the front of the trajectory), while a negative shift value means shifting the trajectory later in time (i.e. adding placeholder frames to the front of the trajectory). The process of calculating the shift align values is detailed in the Data Alignment section. Since the 12-th jump to each target was used as the reference jump, the shift align values of jump numbers 12, 24, and 36 will always be zero. Sample code to apply these shift alignment values can be found in the `load_joint_traj_and_align.m` MATLAB script included in the dataset.

P##_JA_jump_$.csv Located in the `P##_DataJoint Angles` folder corresponding to each participant, this file includes the joint angle trajectories of the kinematic model for the entire jump. The `$` symbol corresponds to the jump number of the participant in jump ordering. A joint angle trajectory for each of the 33 model joints is presented in each column, with the header row names corresponding to each joint. Angles are in radians, with the positive direction being in the same direction as the joint name, and the zero angle reference equal to the zero pose of the model, as described in the Kinematic Model section. Prismatic joint offsets are in meters.

P##_JA_jump_$_R-shldr_L.csv, P##_JA_jump_$_R-shldr_R.csv, P##_JA_jump_$_R-hip_L.csv, P##_JA_jump_$_R-hip_R.csv

Also located in the ##_DataJoint Angles folder are 3D rotation matrix trajectories for the shoulder and hip joints. These rotation transformations describe the shoulder (elevation, abduction, ext. rotation) and hip (flexion, abduction, ext. rotation) rotations, as explained in the Pose Estimation Filtering section. Matrices are presented for each frame of the jump recording in column-major vectorized format as shown below:

$$\begin{bmatrix} a & d & g \\ b & e & h \\ c & f & i \end{bmatrix} = \begin{bmatrix} a & b & c & d & e & f & g & h & i & j & k & l \end{bmatrix}$$

Note that all joint angle trajectories (all data files in P##_DataJoint Angles) are presented in their unaligned state, directly after the pose estimation filtering process. To align the trajectories using our alignment algorithm you must apply the temporal shifts included in P##_shift_align_values.csv, which is provided in sample code.

Technical Validation

The primary metric for validating the pose estimation results was the marker position MAE. The addition of a prismatic joint to the shoulder of the kinematic model, calibration window tuning, and the EKF update model and process noise were all optimised to reduce the average MAE over all markers, jumps and participants.

Marker MAE

The MAE (1) was calculated for each marker, at each frame of a recorded jump, for all performed jumps and for all participants.

Full Body MAE for a Jump

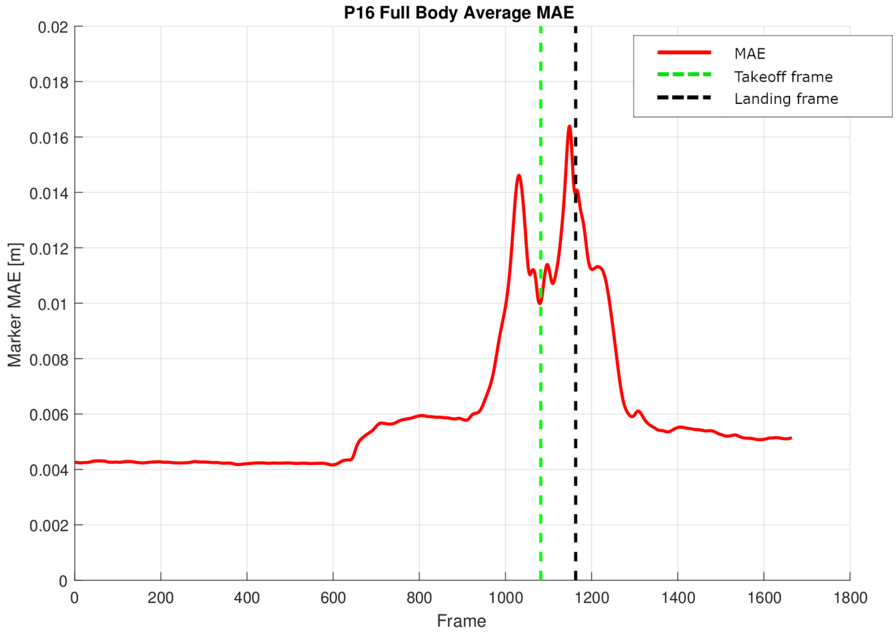

Figure S5. Example full body marker MAE. Here the MAE is averaged over all markers at each frame of the trajectory. The green and black dotted lines indicate the takeoff and landing frames, respectively.

Averaging the MAE over all markers gives the average full body marker error over the course of a single jump. Figure S5 shows a typical full body marker position MAE. The highest MAE occurs at the most dynamic portions of the jump: the takeoff portion and the landing impact.

Overall Average MAE for each Participant

For this analysis, the marker MAE values were averaged over all frames and all of a participant's jumps, resulting in average MAE values for each marker (30) from each participant (22). Table S6 shows these results, as well as the average MAE for all

markers in the last column, and average MAE for each participant in the last row. Different participants have slightly different total average error, usually due to differences in technique or marker attachment conditions.

The lower body markers have the lowest average MAE, likely due to a truer approximation of the lower body kinematics with the model, since it is mostly sagittal plane movement. The less accurate kinematic shoulder model results in higher average MAE in the shoulder and elbow markers. The fact that the hip and back markers were attached to the participant's clothing rather than skin likely contributes to higher MAE for these markers, especially when participants flex their hips before takeoff.

Average Peak MAE for each Participant

For this analysis, the peak marker MAE values were found for each single jump, then these peak values were averaged over all jumps from one participant. Table S7 contains contains the average peak MAE values. It is clear that the lower back markers often show the highest peak errors during a jump, likely due to the approximated hip joint centre model.

Pose Estimation Optimisation

Shoulder Prismatic Joint

We compared two simplified models for the kinematic shoulder joint: The first model consisted of three orthogonal Euler angles, representing the glenohumeral joint, a constant offset distance away from the upperback model frame. In the second model, a prismatic joint was added to the shoulder model, along the frontal axis of the body, to better approximate the motion of the scapula. The average shoulder marker MAE with the first model was higher than all other body markers, especially when the participant lowered their arms and stood straight after the calibration pose. For the second model, the average MAE of the shoulder markers improved by 2.9 mm, with peak improvements of up to 20 mm (while standing straight, arms down). The neck marker MAE improved by 2.1 mm on average, and the MAE of all other body markers had negligible difference.

EKF Update Model

The EKF pose estimation uses a standard constant acceleration update model. A constant velocity update model was also tested on all of the data. The average difference in marker MAE was found to be negligible between these two filters, however, it was found that during the most dynamic parts of the jumping motion the constant velocity model responded more slowly to the change in position.

EKF Process Noise

The observation noise of the EKF pose estimation was set to 0.01, since the average marker MAE is at or below 10 mm. Relative to this observation noise, the process noise value of 1.05 was found to result in the lowest marker MAE for all jumps from all participants. This optimization was computed using MATLAB's nonlinear, constrained minimizer function `fmincon`.

Calibration Window Start Frame and Length

A calibration window was used to calculate the initial positions of the markers relative to the kinematic model frames. This window is placed within the initial 4 second (800 frames) calibration pose at the beginning of each jump recording. A grid search was performed to find the starting frame and length of the calibration window that resulted in the lowest overall MAE for all jumps and participants. A window from frames 250 to 450 was found to have the lowest and most consistent marker MAE for all jumps from all participants, so the kinematic model link lengths and marker attachments are calculated using the marker positions within this window.

Usage Notes

- Data for participant ID 01 are not included in this dataset. This participant did not have previous experience jumping to a target.
- Participant ID 02 used a T-pose calibration, while all other participants used a L-pose calibration.
- The markers used to locate the takeoff line were sometimes put in an opposite order than they were labelled. This means sometimes the START_L marker is on the right (global −Y), and the START_R marker is on the left (global +Y).

Table S6. MAE for each marker [in mm], averaged over all jumps from each participant. The three highest marker MAEs (worst performance) for each participant are highlighted and bolded.

Body Section	Marker Label	Participant ID Number																					Average
		2	3	4	5	6	7	8	9	10	11	12	13	14	15	16	17	18	19	20	21	22	
Upper Back	Neck	**18**	11	9	11	9	7	9	15	10	12	6	9	11	6	8	11	9	8	12	**15**	9	10
	SL	15	12	7	16	9	9	**13**	19	**11**	**13**	8	14	12	9	**11**	11	8	**11**	**19**	12	12	12
	SR	**15**	12	9	**21**	10	9	10	17	10	12	6	15	12	10	8	11	8	9	**17**	12	**14**	12
Pelvis	HL	9	11	7	**16**	11	8	10	21	7	10	6	**17**	**13**	13	**9**	**15**	6	8	15	12	**12**	11
	HR	11	9	8	13	9	8	9	**30**	6	9	6	14	10	11	**11**	**14**	7	10	**20**	**13**	10	11
	BT	9	**15**	**13**	10	10	7	8	**23**	8	11	7	7	9	9	8	11	5	9	14	10	8	10
	BL	8	**17**	12	12	11	8	10	20	6	11	7	9	11	10	9	**12**	5	10	12	10	10	10
	BR	10	**15**	12	12	10	8	10	**24**	6	12	6	10	9	9	9	11	5	10	13	12	9	11
Left Arm	ELLat	15	10	7	10	13	7	11	20	**16**	7	**10**	10	8	16	8	10	**10**	10	11	12	8	11
	ELMed	**15**	9	8	10	**15**	7	10	21	**14**	8	8	13	11	**17**	7	9	9	**13**	13	11	7	11
	WLLat	6	3	4	5	6	3	3	5	4	3	3	6	4	3	3	5	6	5	5	4	2	4
	WLMed	6	4	5	4	3	4	4	8	6	5	4	6	4	6	5	4	6	7	8	5	6	5
Right Arm	ERLat	6	11	**14**	**17**	**14**	9	7	17	11	12	**15**	**18**	**16**	9	5	10	9	10	13	**13**	**12**	12
	ERMed	7	10	12	14	**14**	**10**	10	17	10	**13**	**14**	**17**	**16**	12	6	10	10	**12**	10	11	11	12
	WRLat	4	4	4	3	4	2	3	6	5	3	4	7	5	3	2	6	3	4	6	4	4	4
	WRMed	5	4	4	6	3	5	3	6	5	5	4	5	5	7	4	4	3	5	6	6	6	5
Left Leg	KLLat	14	11	10	9	11	10	**12**	20	7	9	6	8	7	9	6	8	**11**	8	10	7	6	9
	KLMed	10	10	9	8	9	**12**	**13**	12	7	8	6	6	7	9	5	7	**13**	10	8	8	6	9
	ALLat	6	5	5	6	4	5	6	12	5	5	4	5	5	7	6	6	5	5	7	6	4	6
	ALMed	5	6	4	7	5	4	6	9	4	4	4	7	6	6	5	6	4	5	8	8	5	6
	FLLat	3	7	3	8	5	3	5	9	2	5	3	10	6	5	6	8	4	4	9	6	6	5
	FLMed	4	4	3	5	3	2	4	11	2	3	3	5	4	3	5	5	3	3	6	7	4	4
	FLHeel	7	6	6	7	6	4	5	12	4	5	5	7	5	6	5	6	4	5	8	7	5	6
Right Leg	KRLat	11	12	**15**	9	9	8	11	21	7	**13**	6	6	8	**18**	5	9	8	7	6	6	6	10
	KRMed	9	9	11	10	7	**10**	11	14	8	10	5	4	8	**20**	6	6	9	10	6	10	5	9
	ARLat	5	7	6	6	5	4	6	9	5	5	3	7	5	8	5	7	4	5	6	5	6	6
	ARMed	6	6	4	7	4	4	5	8	4	4	3	8	6	7	5	6	4	4	9	6	4	5
	FRLat	5	5	3	8	4	2	4	12	2	4	2	9	6	5	6	6	3	3	10	6	5	5
	FRMed	4	4	3	5	3	2	3	10	2	4	2	6	4	5	5	6	3	2	6	5	5	4
	FRHeel	6	6	5	6	6	5	6	9	4	7	4	8	6	5	6	6	5	5	7	7	6	6
	**Average**	9	9	7	9	8	6	8	15	7	8	6	9	8	9	6	8	6	7	10	8	7	

Table S7. Maximum MAE for each marker per jump [in mm], averaged over all jumps from each participant. The three highest marker MAE peaks (worst performance) for each participant are highlighted and bolded.

Body Section	Marker Label	Participant ID Number																					Average
		2	3	4	5	6	7	8	9	10	11	12	13	14	15	16	17	18	19	20	21	22	
Upper Back	Neck	**56**	29	27	33	29	22	28	47	**37**	42	17	30	**37**	26	24	34	**29**	28	37	**51**	37	33
	SL	32	32	30	41	25	24	**52**	65	**38**	27	21	**31**	**39**	27	31	31	29	37	**54**	21	27	34
	SR	34	31	29	**56**	24	30	40	55	**39**	28	28	**31**	35	39	19	44	28	23	35	21	39	34
Pelvis	HL	26	28	29	**45**	32	28	29	39	27	32	36	**33**	31	47	18	31	25	26	26	25	33	31
	HR	30	22	33	27	29	29	28	59	25	27	30	23	24	33	28	25	28	31	**43**	32	24	30
	BT	**35**	**42**	**48**	40	**40**	**38**	37	**69**	31	**49**	**47**	26	35	**49**	**33**	**46**	29	**39**	**42**	44	**42**	41
	BL	35	**46**	**46**	**42**	**44**	**42**	**43**	**65**	29	**47**	**48**	28	**40**	**52**	**35**	**46**	**30**	**43**	37	**44**	**42**	42
	BR	**37**	**42**	**47**	42	**37**	**44**	**44**	**67**	32	**54**	**43**	25	34	**49**	**36**	**45**	**32**	**42**	38	**46**	**41**	42
Left Arm	ELLat	22	26	15	16	18	16	30	36	22	16	16	18	20	22	19	23	20	18	28	19	16	21
	ELMed	21	24	17	17	25	15	29	42	23	19	16	23	21	25	19	23	21	24	31	17	17	22
	WLLat	11	16	7	11	12	9	9	19	10	15	7	18	12	8	8	20	12	9	12	7	9	12
	WLMed	14	15	12	11	7	14	19	25	12	17	11	14	13	14	19	17	12	16	20	14	19	15
Right Arm	ERLat	15	19	20	29	21	15	19	30	19	19	19	23	26	21	10	30	17	19	25	21	22	21
	ERMed	16	21	20	24	19	18	31	30	20	21	17	27	26	27	12	26	19	23	23	16	21	22
	WRLat	11	14	9	8	10	7	11	17	7	9	6	12	13	8	7	17	12	8	20	7	13	11
	WRMed	11	15	10	17	7	21	8	18	12	17	7	10	14	21	11	13	9	9	18	15	23	14
Left Leg	KLLat	35	30	33	25	29	18	19	59	24	29	33	22	23	34	25	30	18	31	34	31	21	29
	KLMed	34	39	27	22	30	28	22	40	35	27	26	14	27	29	20	34	27	24	33	28	21	28
	ALLat	17	17	20	21	17	16	15	29	14	23	19	14	15	19	20	16	14	17	19	14	14	18
	ALMed	17	17	11	18	18	14	16	20	18	15	11	12	15	18	12	17	14	14	18	16	11	15
	FLLat	15	12	11	12	12	9	22	18	12	10	17	13	16	14	14	14	18	13	23	20	11	14
	FLMed	11	11	10	10	11	9	14	14	10	10	17	15	13	14	13	13	10	11	13	13	10	12
	FLHeel	21	22	25	24	27	20	18	35	13	23	28	20	20	24	23	19	19	23	20	22	23	22
Right Leg	KRLat	30	35	35	32	29	20	26	46	20	32	33	21	22	40	20	30	16	18	25	16	23	27
	KRMed	20	34	34	24	28	26	27	40	34	31	18	16	24	32	21	29	25	27	23	30	22	27
	ARLat	14	22	21	22	15	19	14	21	16	23	17	13	13	18	16	15	13	14	15	13	17	17
	ARMed	14	18	13	13	13	13	16	22	22	10	10	10	12	15	12	14	12	10	21	14	10	14
	FRLat	12	14	13	19	12	9	19	18	14	15	18	20	19	21	11	22	19	8	14	12	16	15
	FRMed	11	12	13	12	11	10	17	18	15	14	13	14	13	16	9	11	14	8	11	14	12	13
	FRHeel	18	22	27	23	22	21	19	36	17	24	24	15	14	22	22	16	18	19	17	22	21	21
	**Average**	23	24	23	24	22	20	24	37	22	24	22	20	22	26	19	25	20	21	26	22	22	

Supplementary Figures and Tables

This section contains supplementary tables and figures. Table S8 provides a breakdown of each participant analyzed, their jump performance and style, as well as hours of exercise per week. Figure S6 contains a set of sample CoM trajectories from a participant. Figures S7, S8, S9, and S10 show the 4 different jumping styles in IOC recovered weights.

Table S8. Participant statistics from jumping experiment. Expertise is labeled as novice (N) or expert (E), as classified by their experimental jump success. Experts are also denoted by green rows. Participants marked with a ! had self-declared previous experience with jumping to a target.

Participant Number	Number of Jumps for Each Grade						Expertise	Jump Style	Exercise [hrs/week]
	B	SB	P	P*	SF	F			
2	0	2	22	7	5	0	E	C	2
3	0	4	10	12	5	5	N	-	3
4	0	2	16	7	3	8	N	C	6
5	0	4	14	12	5	1	N	B	0
6	0	0	20	9	5	2	N	B	4
7	0	4	29	3	0	0	E	D	1
8	0	3	22	11	0	0	E	A	3
9	0	3	19	8	3	3	N	D	6
10 !	1	0	32	3	0	0	E	A	8
11	1	2	18	5	4	6	N	A	6
12	2	0	32	2	0	0	E	D	9
13	2	8	19	5	0	2	N	-	2
14	1	6	15	10	4	0	N	A	5
15	0	1	20	8	5	2	N	D	0
16	1	3	26	3	3	0	N	B	7
17 !	1	4	29	2	0	0	E	C	15
18	1	1	21	8	4	1	N	D	0
19	1	7	21	6	0	1	N	-	6
20 !	0	2	30	4	0	0	E	C	20
21	4	6	21	3	1	1	N	B	7
22	1	2	25	7	1	0	E	A	3
Total	16	64	461	135	48	32			
Mean Expert	0.7	2.2	27.4	4.7	1.0	0.0			
Mean Novice	0.8	3.7	17.8	7.8	3.3	2.7			

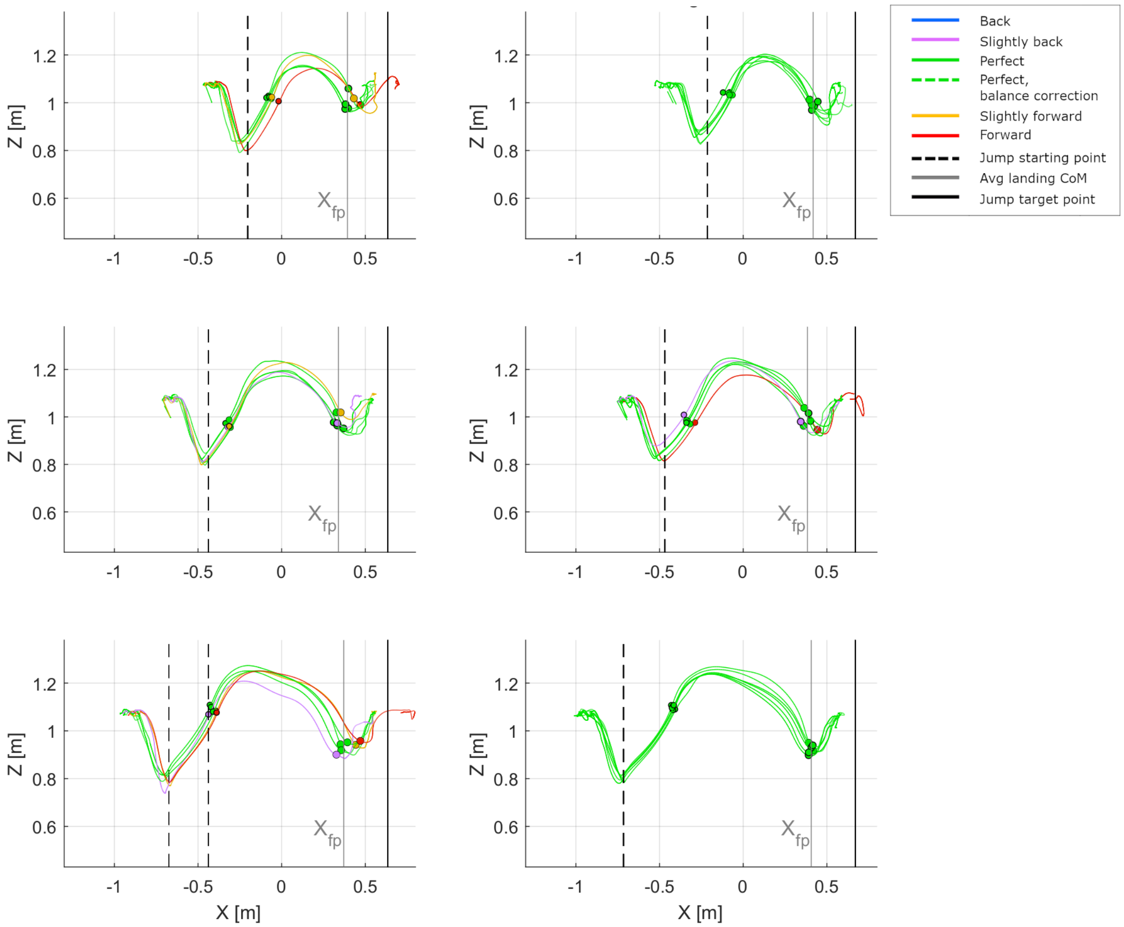

Figure S6. CoM trajectories from all jumps performed by Participant 9. Foot placement pose clustering (circles on the right side of each plot, marking CoM position at landing) is seen to improve in the second set of jumps (right column) to each target, especially for the short and long targets (top and bottom rows, respectively).

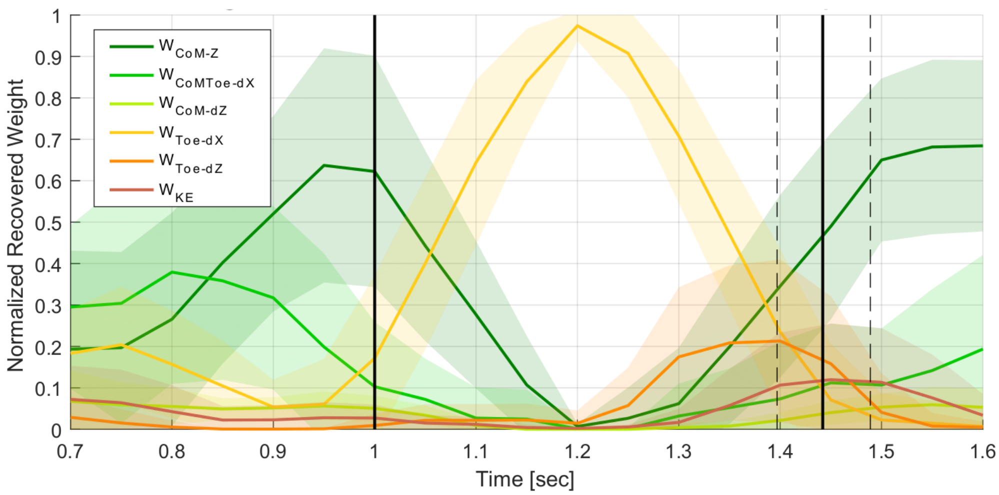

Figure S7. Mean weight trajectories for jumping style group A, controlled takeoff and landing.

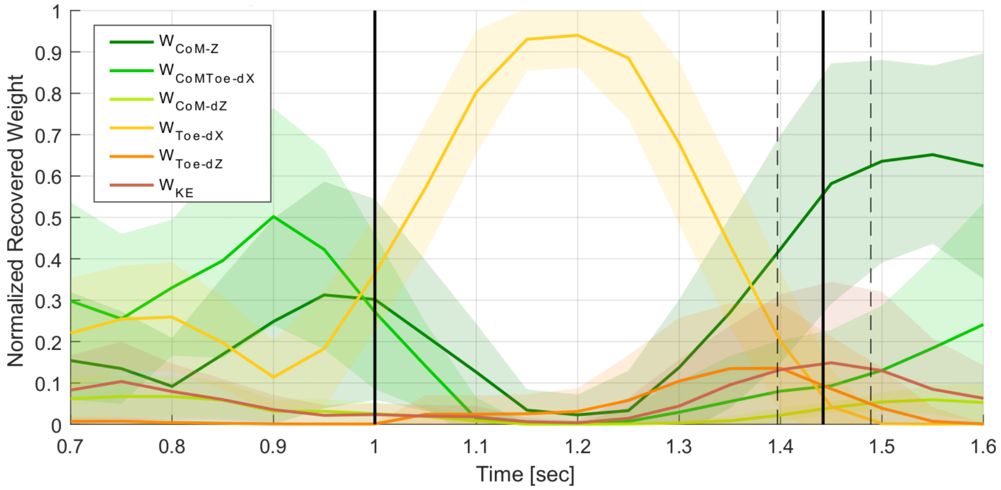

Figure S8. Mean weight trajectories for jumping style group B, stiff legged landing.

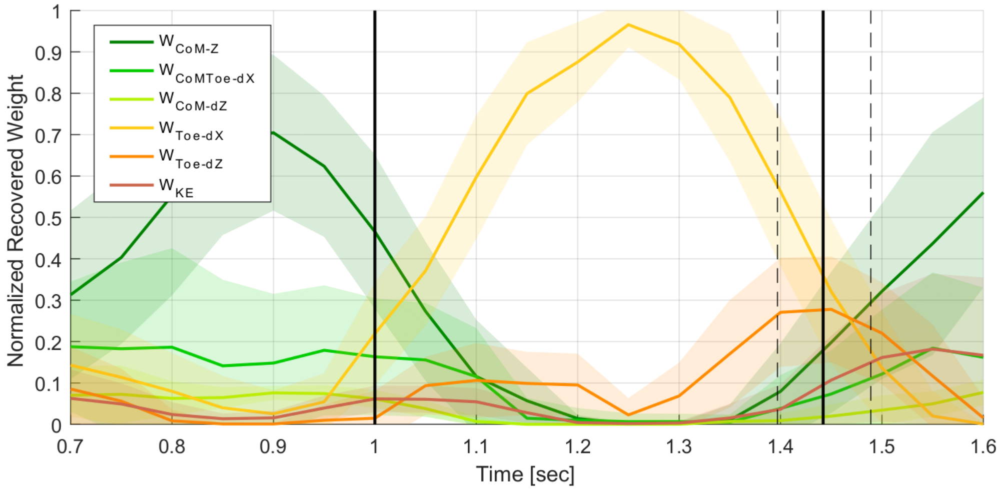

Figure S9. Mean weight trajectories for jumping style group C, explosive takeoff.

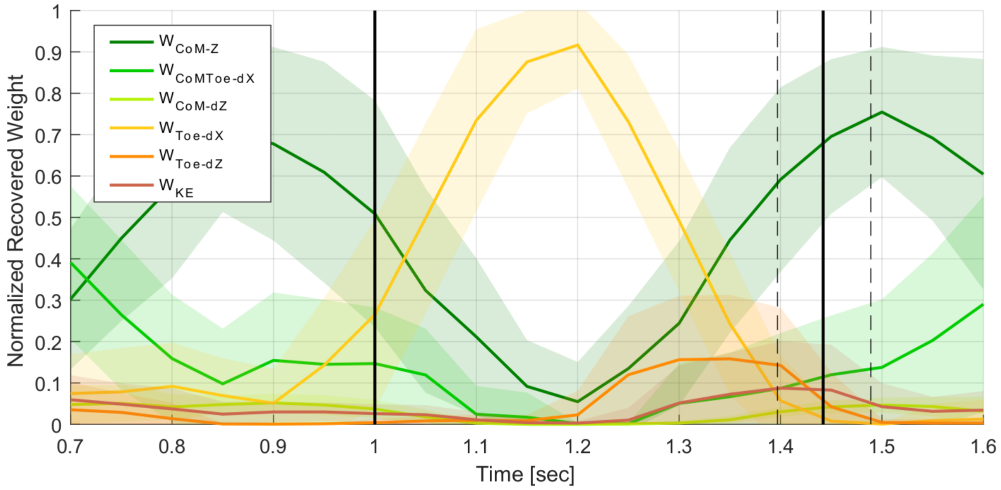

Figure S10. Mean weight trajectories for jumping style group D, hesitant landing.

Supplementary Statistical Analysis

This section contains the input and output details of the statistics that were performed in this paper. All statistical analysis was done using MATLAB 9.6 and utilised either the repeated measures (RM) ANOVA (`ranova`) or the logistic regression (`mnrfit`). In all tables listed in this section, the bolded values are quoted in the main manuscript.

In Section 4.3, RM ANOVA was used to assess if the jump grade differed between jump set 1 and jump set 2, suggesting that motor learning occurred and improved the jump grades in jump set 2. Participant expertise was the between subject variable while jump set and jump distance was the within subject variable. The MATLAB output of this analysis is as follows:

	SumSq	DF	MeanSq	F	pValue
(Intercept)	292.19	1	292.19	646.56	3.8942e-16
Expert	11.551	1	11.551	25.561	7.0197e-05
Error	8.5863	19	0.45191		
(Intercept):JumpDist	1.3719	2	0.68595	2.726	0.078289
Expert:JumpDist	0.57825	2	0.28912	1.149	0.32772
Error(JumpDist)	9.562	38	0.25163		
**(Intercept):JumpSet**	**278.67**	**1**	**278.67**	**675.81**	**2.5864e-16**
Expert:JumpSet	10.919	1	10.919	26.48	5.7452e-05
Error(JumpSet)	7.8345	19	0.41234		
(Intercept):JumpDist:JumpSet	1.5848	2	0.79241	3.3903	0.044177
Expert:JumpDist:JumpSet	0.59647	2	0.29823	1.276	0.29085
Error(JumpDist:JumpSet)	8.8818	38	0.23373		

In Section 4.4, RM ANOVA was used to assess if the variance of the takeoff and landing angles differed between experts and novices, in order to assess an impact of expertise on kinematic measures. The variance of the 36 takeoff and landing angles were calculated, separated by jump set and distance, resulting in 6 angle variance values. Participant expertise was the between subject variable while jump set and jump distance was the within subject variable. The MATLAB output of the takeoff angle analysis is as follows:

	SumSq	DF	MeanSq	F	pValue
(Intercept)	18011	1	18011	15.913	0.0007856
**Expert**	**2940.1**	**1**	**2940.1**	**2.5975**	**0.12352**
Error	21506	19	1131.9		
(Intercept):JumpDist	3672.7	2	1836.4	1.5678	0.22169
Expert:JumpDist	507.25	2	253.63	0.21653	0.80629
Error(JumpDist)	44509	38	1171.3		
(Intercept):JumpSet	29.479	1	29.479	0.035417	0.85272
Expert:JumpSet	47.839	1	47.839	0.057477	0.8131
Error(JumpSet)	15814	19	832.32		
(Intercept):JumpDist:JumpSet	160.74	2	80.369	0.08629	0.91751
Expert:JumpDist:JumpSet	226.08	2	113.04	0.12137	0.88605
Error(JumpDist:JumpSet)	35392	38	931.38		

The MATLAB output of the landing angle analysis is as follows:

	SumSq	DF	MeanSq	F	pValue
(Intercept)	3.2584e+05	1	3.2584e+05	17.246	0.0005404
**Expert**	**1.0578e+05**	**1**	**1.0578e+05**	**5.5991**	**0.02875**
Error	3.5897e+05	19	18893		
(Intercept):JumpDist	3.12e+05	1	3.12e+05	17.075	0.00056656
Expert:JumpDist	98580	1	98580	5.395	0.031451

Error(JumpDist)	3.4718e+05	19	18272		
(Intercept):JumpSet	2.6429e+05	1	2.6429e+05	18	0.00044034
Expert:JumpSet	83232	1	83232	5.6686	0.02789
Error(JumpSet)	2.7898e+05	19	14683		
(Intercept):JumpDist:JumpSet	2.5334e+05	1	2.5334e+05	18.53	0.00038231
Expert:JumpDist:JumpSet	77242	1	77242	5.6496	0.028122
Error(JumpDist:JumpSet)	2.5977e+05	19	13672		

In Section 5.1, RM ANOVA was to assess if cost weights differ with respect to jumping distance. Participant expertise was the between subject variable while jump set and jump distance was the within subject variable. The MATLAB output of the w_{CoM-Z} cost weight on takeoff is as follows:

	SumSq	DF	MeanSq	F	pValue
(Intercept)	156.79	1	156.79	122.32	1.0143e-09
Expert	1.0747	1	1.0747	0.83848	0.37131
Error	24.354	19	1.2818		
**(Intercept):JumpDist**	**0.86883**	**2**	**0.43442**	**4.2559**	**0.021488**
Expert:JumpDist	0.19762	2	0.098809	0.96801	0.38901
Error(JumpDist)	3.8788	38	0.10207		
(Intercept):JumpSet	1.0494	1	1.0494	18.061	0.00043322
Expert:JumpSet	0.023816	1	0.023816	0.40988	0.52967
Error(JumpSet)	1.104	19	0.058104		
(Intercept):JumpDist:JumpSet	0.061335	2	0.030667	0.4804	0.62224
Expert:JumpDist:JumpSet	0.46999	2	0.23499	3.6812	0.034567
Error(JumpDist:JumpSet)	2.4258	38	0.063837		

The MATLAB output of the w_{CoM-Z} cost weight on landing is as follows:

	SumSq	DF	MeanSq	F	pValue
(Intercept)	263.06	1	263.06	297.73	4.5764e-13
Expert	2.6222	1	2.6222	2.9679	0.10117
Error	16.787	19	0.88352		
**(Intercept):JumpDist**	**3.9118**	**2**	**1.9559**	**18.78**	**2.1297e-06**
Expert:JumpDist	0.091259	2	0.045629	0.43812	0.64846
Error(JumpDist)	3.9576	38	0.10415		
(Intercept):JumpSet	0.069595	1	0.069595	2.0911	0.16446
Expert:JumpSet	0.00028795	1	0.00028795	0.0086519	0.92686
Error(JumpSet)	0.63236	19	0.033282		
(Intercept):JumpDist:JumpSet	0.094888	2	0.047444	1.7082	0.19482
Expert:JumpDist:JumpSet	0.094164	2	0.047082	1.6951	0.19716
Error(JumpDist:JumpSet)	1.0554	38	0.027775		

The MATLAB output of the $w_{CoMToe-dX}$ cost weight on landing is as follows:

	SumSq	DF	MeanSq	F	pValue
(Intercept)	1.2777	1	1.2777	12.923	0.0019312
Expert	0.18836	1	0.18836	1.9051	0.18353
Error	1.8786	19	0.098871		
**(Intercept):JumpDist**	**0.24296**	**2**	**0.12148**	**5.394**	**0.0086687**
Expert:JumpDist	0.050336	2	0.025168	1.1175	0.3376

Error(JumpDist)	0.85581	38	0.022521		
(Intercept):JumpSet	0.00039995	1	0.00039995	0.031352	0.86133
Expert:JumpSet	0.013019	1	0.013019	1.0206	0.32508
Error(JumpSet)	0.24238	19	0.012757		
(Intercept):JumpDist:JumpSet	0.037738	2	0.018869	1.9524	0.15591
Expert:JumpDist:JumpSet	0.053604	2	0.026802	2.7733	0.075123
Error(JumpDist:JumpSet)	0.36725	38	0.0096644		

The MATLAB output of the w_{KE} cost weight on landing is as follows:

	SumSq	DF	MeanSq	F	pValue
(Intercept)	7.1389	1	7.1389	39.926	4.5944e-06
Expert	0.22326	1	0.22326	1.2486	0.27775
Error	3.3973	19	0.1788		
**(Intercept):JumpDist**	**0.90608**	**2**	**0.45304**	**14.888**	**1.6806e-05**
Expert:JumpDist	0.019923	2	0.0099617	0.32737	0.72283
Error(JumpDist)	1.1563	38	0.03043		
(Intercept):JumpSet	0.051246	1	0.051246	2.2077	0.15373
Expert:JumpSet	0.019338	1	0.019338	0.83307	0.37282
Error(JumpSet)	0.44104	19	0.023213		
(Intercept):JumpDist:JumpSet	0.045727	2	0.022864	2.5573	0.090785
Expert:JumpDist:JumpSet	0.013477	2	0.0067386	0.75372	0.47752
Error(JumpDist:JumpSet)	0.33974	38	0.0089404		

Later in Section 5.1, logistic regression was used to assess if cost weights differ if the participant undershot or overshot the target, with respect to landing on the target perfectly. The MATLAB statistical output of the cost weights examined is as follows:

		t-stat	p-val	dof
(Intercept)	Behind	-2.79	0.01	1502
	Forward	-4.84	0.00	1502
**CoM-Z**	**Behind**	**-0.55**	**0.58**	**1502**
	**Forward**	**2.06**	**0.04**	**1502**
**Toe-dZ**	**Behind**	**-1.93**	**0.05**	**1502**
	**Forward**	**1.02**	**0.31**	**1502**

The last statistical analysis performed in Section 5.1 used the RM ANOVA to assess if cost weight differ between experts and novices. Participant expertise was the between subject variable while jump set and jump distance was the within subject variable. The MATLAB output of the w_{CoM-Z} cost weight on landing is as follows:

	SumSq	DF	MeanSq	F	pValue
(Intercept)	263.06	1	263.06	297.73	4.5764e-13
**Expert**	**2.6222**	**1**	**2.6222**	**2.9679**	**0.10117**
Error	16.787	19	0.88352		
(Intercept):JumpDist	3.9118	2	1.9559	18.78	2.1297e-06
Expert:JumpDist	0.091259	2	0.045629	0.43812	0.64846
Error(JumpDist)	3.9576	38	0.10415		
(Intercept):JumpSet	0.069595	1	0.069595	2.0911	0.16446
Expert:JumpSet	0.00028795	1	0.00028795	0.0086519	0.92686
Error(JumpSet)	0.63236	19	0.033282		
(Intercept):JumpDist:JumpSet	0.094888	2	0.047444	1.7082	0.19482
Expert:JumpDist:JumpSet	0.094164	2	0.047082	1.6951	0.19716
Error(JumpDist:JumpSet)	1.0554	38	0.027775		

In Section 5.2, RM ANOVA was used to assess if cost weight differ between jump styles, as determined by clustering analysis. Unlike previous sections, only participants with a labelled jump style was included in the analysis, leading to 3 participants being excluded from this analysis (Table S8). Each participant/distance combination was considered independent since it was observed that different jump distances may have some effort on resultant jump style. Overall participant jump style was the between subject variable while jumpset was the within subject variable. The MATLAB output of the w_{CoM-Z} cost weight on takeoff is as follows:

	SumSq	DF	MeanSq	F	pValue
(Intercept)	152.41	1	152.41	385.71	3.7154e-25
**JumpStyle**	**7.9884**	**3**	**2.6628**	**6.739**	**0.00066082**
Error	19.757	50	0.39513		
(Intercept):JumpSet	1.0201	1	1.0201	15.084	0.00030261
JumpStyle:JumpSet	0.35806	3	0.11935	1.7648	0.1659
Error(JumpSet)	3.3815	50	0.06763		

The MATLAB output of the w_{CoM-Z} cost weight on landing is as follows:

	SumSq	DF	MeanSq	F	pValue
(Intercept)	235.15	1	235.15	1028.9	5.1246e-35
**JumpStyle**	**9.9503**	**3**	**3.3168**	**14.513**	**6.3177e-07**
Error	11.427	50	0.22854		
(Intercept):JumpSet	0.067267	1	0.067267	2.2048	0.14386
JumpStyle:JumpSet	0.18664	3	0.062212	2.0391	0.1203
Error(JumpSet)	1.5255	50	0.03051		

The MATLAB output of the maximum $w_{CoMToe-dX}$ cost weight over the full trajectory is as follows:

	SumSq	DF	MeanSq	F	pValue
(Intercept)	608.1	1	608.1	81673	5.2002e-82
**JumpStyle**	**0.066144**	**3**	**0.022048**	**2.9612**	**0.040988**
Error	0.37228	50	0.0074455		
(Intercept):JumpSet	9.0783e-05	1	9.0783e-05	0.14932	0.70082
JumpStyle:JumpSet	0.0062386	3	0.0020795	3.4205	0.024146
Error(JumpSet)	0.030398	50	0.00060796		

The MATLAB output of the w_{Toe-dZ} cost weight on landing is as follows:

	SumSq	DF	MeanSq	F	pValue
(Intercept)	4.54	1	4.54	112.91	2.0032e-14
**JumpStyle**	**4.4657**	**3**	**1.4886**	**37.021**	**9.5535e-13**
Error	2.0104	50	0.040208		
(Intercept):JumpSet	0.051301	1	0.051301	4.6821	0.035281
JumpStyle:JumpSet	0.026094	3	0.0086979	0.79384	0.5031
Error(JumpSet)	0.54783	50	0.010957		

The MATLAB output of the w_{KE} cost weight on landing is as follows:

	SumSq	DF	MeanSq	F	pValue
(Intercept)	5.926	1	5.926	77.069	1.0639e-11
**JumpStyle**	**0.55961**	**3**	**0.18654**	**2.426**	**0.076455**
Error	3.8446	50	0.076892		
(Intercept):JumpSet	0.044563	1	0.044563	4.5379	0.038097
JumpStyle:JumpSet	0.2037	3	0.0679	6.9143	0.00055274
Error(JumpSet)	0.49101	50	0.0098202		

References

1. Harrington, M., Zavatsky, A., Lawson, S., Yuan, Z. & Theologis, T. Prediction of the hip joint centre in adults, children, and patients with cerebral palsy based on magnetic resonance imaging. *J. Biomech.* **40**, 595–602, DOI: 10.1016/j.jbiomech.2006.02.003 (2007).
2. Joukov, V., D'Souza, R. & Kulić, D. Human pose estimation from imperfect sensor data via the extended kalman filter. In *Int. Symp. Exp. Robot.*, 789–798 (2016).
